# Preparation of GaN/Porous silicon heterojunction photodetector by laser deposition technique

**DOI:** 10.1038/s41598-023-41396-8

**Published:** 2023-09-07

**Authors:** Makram A. Fakhri, Haneen D. Jabbar, Mohammed Jalal AbdulRazzaq, Evan T. Salim, Ahmad S. Azzahrani, Raed Khalid Ibrahim, Raid A. Ismail

**Affiliations:** 1 Applied Science Department, University of Technology-Iraq, Baghdad, Iraq; 2 Laser and Optoelectronic Engineering Department, University of Technology-Iraq, Baghdad, Iraq; 3 Electrical Engineering Department, Northern Border University, Arar, Kingdom of Saudi Arabia; 4 AlFarahidi University, Baghdad, Iraq

**Keywords:** Materials science, Nanoscience and technology, Optics and photonics

## Abstract

In this work, gallium nitride (GaN) thin film was deposited on porous silicon (PSi) substrate via a pulsed laser deposition route with a 355 nm laser wavelength, 900 mJ of laser energy, and various substrate temperatures raging from 200 to 400 °C. The structural and optical properties of GaN films as a function of substrate temperature are investigate. XRD studies reveal that the GaN films deposited on porous silicon are nanocrystalline with a hexagonal wurtzite structure along (002) plane. The photoluminescence emission peaks of the GaN/PSi prepared at 300 °C substrate temperature are located at 368 nm and 728 nm corresponding to energy gap of 3.36 eV and 1.7 eV, respectively. The GaN/PSi heterojunction photodetector prepared at 300 °C exhibits the maximum performance, with a responsivity of 29.03 AW^−1^, detectivity of 8.6 × 10^12^ Jones, and an external quantum efficiency of 97.2% at 370 nm. Similarly, at 575 nm, the responsivity is 19.86 AW^−1^, detectivity is 8.9 × 10^12^ Jones, and the external quantum efficiency is 50.89%. Furthermore, the photodetector prepared at a temperature of 300 °C demonstrates a switching characteristic where the rise time and fall time are measured to be 363 and 711 μs, respectively.

## Introduction

There are numerous uses for ultraviolet photodiodes, including environmental monitoring, optical communication, the detection of missiles, and space exploration^1–4^. In order to produce a p–n junction photodiode, it is necessary to select a wavelength that minimises the background noise generated by the remainder of the spectrum^5–8^ as well as construct the photodiode's structure from a material that determines its adaptability in severe environments^9–12^. In modern optoelectronics, the p–n junction photodiode is a fundamental component that is made by combining two semiconductor materials with different bandgaps and other properties, opening the door to novel functionalities and improved overall performance of optoelectronic devices (laser diodes, light-emitting diodes (LED), solar cells, and photodiodes)^13–18^.

The next generation of photodiodes is expected to exhibit increased light absorption, photo-responsivity, and spectrum sensitivity^19–22^. As a result, gallium nitride (GaN) stands out as the foundational material for the nitride class of III-nitride semiconductor materials, thanks to its superior thermodynamic stability^23–25^. GaN is characterized by excellent thermal stability, a small dielectric constant, high thermal conductivity, chemical inertness, radiation hardness, and a wide direct band gap of 3.4 eV^26–30^. Moreover, GaN possesses exceptional UV photoresponse, well-established mixing techniques, and the capability to operate effectively in high-temperature and challenging environments. Consequently, it finds widespread use in optoelectronic devices that necessitate a layer enabling fast carrier transport and a high breakdown voltage. GaN is particularly utilized in the fabrication of high-power and high-temperature devices operating in the blue and ultraviolet wavelengths^31–33^.

Pulsed laser deposition (PLD) is capable of producing high-quality GaN thin films at lower growth temperatures compared to other deposition techniques like MOCVD and MBE^34^. By adjusting laser parameters such as fluence, pulse duration, and distance between target and substrate, stoichiometry of the deposited films can be precisely controled. Consequently, PLD enables the deposition of GaN films with fewer defects and improved optical and electrical characteristics^35^. High-power pulsed lasers facilitate high deposition rates, leading to faster GaN film growth. Furthermore, PLD allows for the deposition of GaN thin films on temperature- sensitive substrates like plastics and polymers at low temperatures. It generates a highly focused and confined plume of materials, resulting in reduced contamination during the deposition process^36,37^. Additionally, the PLD is highly accurate and does not require extensive monitoring since the composition of the film replicates the composition of the target. The background gas pressure has no impact on the passage or absorption of the laser beam in PLD, allowing the same system to be utilized for growing thin film consisting of various materials. This is achieved by adjusting the background gas pressure and positioning different targets under the laser beam^38^.

Furthermore, PLD has the capability to create layered materials by utilizing a computer-controlled multi-target holder or carousel^39^. The ablation geometry provides a degree of freedom as the laser energy source is focused outside the vacuum chamber. This allows for rapid transmission of the target material, leading to increased stoichiometry control increase and significantly faster adjustment of the deposition conditions during the initial tuning process. This feature is particularly advantageous when conducting experiments with different target compositions^40^.

Finally, PLD can be performed on silicon, sapphire, and glass^41^. Among these options, the Si (silicon) substrates have garnered significant attention from researchers due to several advantages, including their cost-effectiveness, good thermal conductivity, ease of fabrication, and abiity to provide uniform carrier injection into the device. However, Si substrates have a notable mismatch in lattice constant with GaN, leading to high defects density and occurrence of cracks^42,43^. To address this issue, many researchers have explored the deposition of an AlN buffer layer prior to GaN development in order to reduce flaws and cracks in subsequent layers. Nevertheless, the formation of an AlN buffer layer is time-consuming and expensive, and it may impede carrier injection within the nitride structure^44–46^. The relationship between the substrate type and the piezoelectric field has been reported^47^. The quantum confined Stark effect (QCSE) arises from the piezoelectric field strength in strained GaN quantum wells. This piezoelectric field strength reduces for GaN when it is deposited on a silicon substrate compared to other substrates^48^.

The objective of this research is to fabricate a high-responsivity photodetector in the UV-A region using a PSi (porous silicon) substrate, in contrast to previous studies that utilized Si and Sapphire substrates. Additionally, this manuscript builds upon our previous work on fabricating porous silicon substrates through a photo-electrochemical etching method, assisted by laser, under optimized etching conditions (10 mA/cm^2^ current density and 10 min etching time)^49,50^.

Furthermore, this manuscript serves as a continuation of our efforts to fabricate GaN/PSi heterojunction photodiodes using the PLD method, exploring different laser wavelengths and energies to identify the optimal growth conditions specifically for a 355 nm laser wavelength and 900 mJ laser energy^51,52^. In this study, GaN/PSi heterojunction photodiodes were grown using a PLD system at various substrate temperatures (ranging from 200 to 400 °C) with an Nd: YAG laser operating at a wavelength of 355 nm and a laser energy of 900 mJ. The influence of variable substrate temperatures on the structural characteristics, optical properties, morphological and topographical features, as well as the electrical and performance behavior, was investigated.

## Experimental

### Preparation of PSi Substrate

The present study extends our previous research in which we utilized porous silicon (PSi) as a substrate to fabricate a GaN/PSi heterojunction photodiode under optimal conditions based on the findings of Haneen et al.^44,49^. The incorporation of a PSi substrate enhances the efficiency of the photodiode through increased surface area and reduced light reflection, resulting in improved light absorption. Furthermore, the porous nature of PSi enables dopant diffusion and the formation of nanostructures, offering opportunities to customize the electronic properties of the material. After establishing the optimal operating conditions, we proceeded to investigate the impact of different laser wavelengths and powers on the deposition process, following the methodology outlined by Haneen et al.^44,49^. This study focuses on the influence of varying substrate temperatures ranging from 200 to 400 °C during the PLD process for growing GaN on a PSi substrate using a 355 nm, 900 mJ Nd: YAG laser. Moreover, we carefully examined the effects on the structural, topographical, morphological, optical, electrical, and performance properties to identify the most favorable substrate temperature condition for achieving optimal growth of GaN on the PSi substrate.

**Figure 1:**
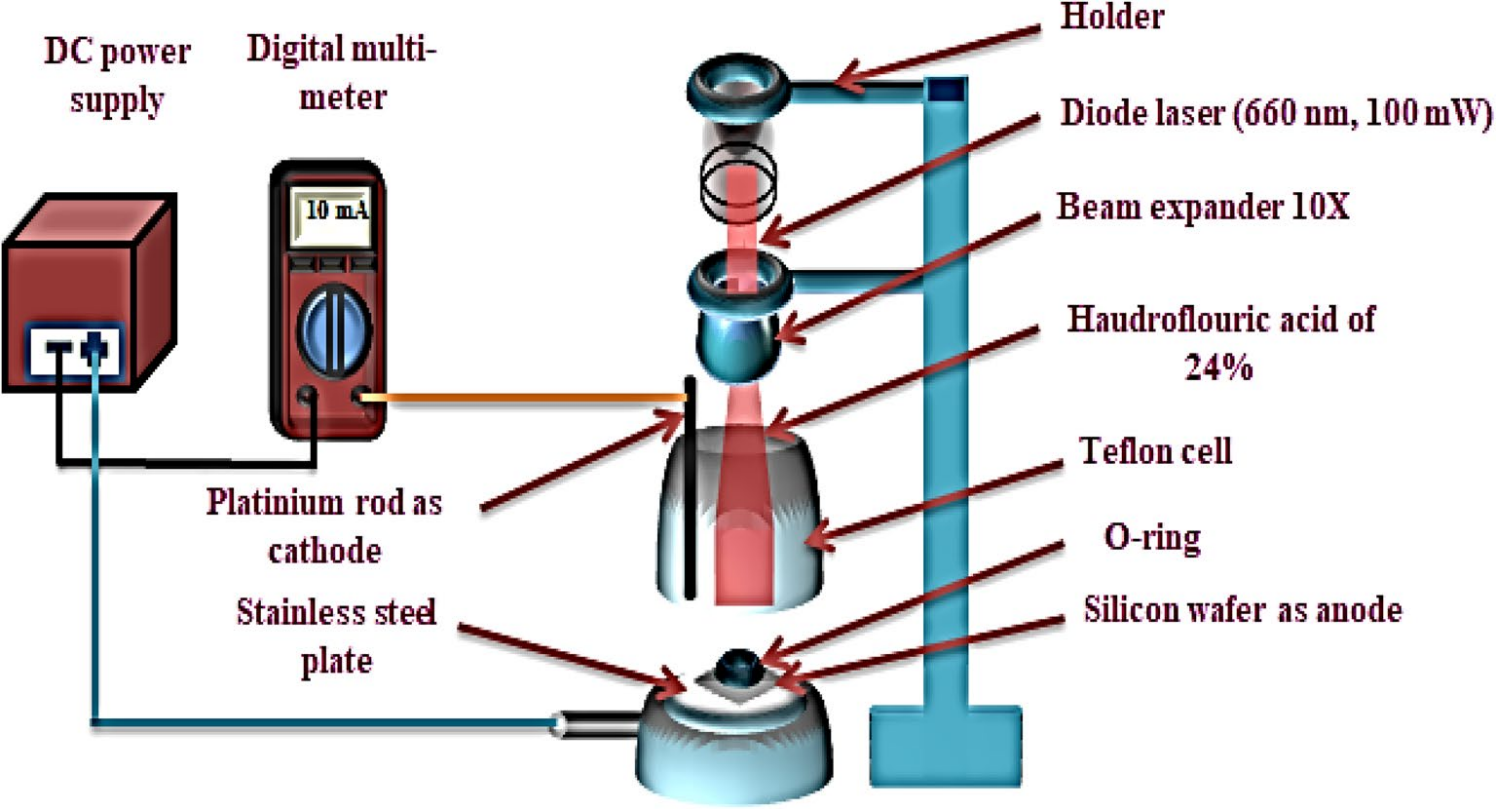
Schematic diagram of Photoelectrochemical etching method assisted by a diode laser under optimum etching conditions^42^.

Mirror-like n-type Si wafers with electrical resistivity of 1–5 mΩ/cm, 500 μm thickness, <100> orientation purchased from University Wafer, Inc, USA were used. The wafers were then cut into rectangular pieces measuring 1 by 1 cm. Prior to the photo-electrochemical etching process, the pieces were thoroughly cleaned using an ultrasonic device in ethanol (99.9% concentration, Germen, Honeywell company) for a duration of 5 min. The etching process took place at room temperature and involved the use of a diode laser (660 nm, 100 mW, China, Tongtool Company), a DC power supply ranging from 0 to 30 V (China, Jiuyuan), and a digital multi-meter (China, Victor). This process, as illustrated in Fig. 1, necessitates the utilization of a Teflon cell equipped with a cathode electrode composed of 95% pure platinum and an anode electrode made of silicon. The laser was an integral part of the top-down electrochemical etching procedure employed to synthesize the PSi substrates. Furthermore, the etching conditions were carefully regulated, with the etching time set at 10 min, the current density maintained at 10 mA/cm^2^, and the concentration of hydrofluoric acid (HF) (obtained from Germen, Thomas Baker company) in the process consistently maintained at 24% using the dilution formula described in Eq. (1)^53–55^.

1 $${\text{C}}_{1} {\text{V}}_{1} = {\text{C}}_{2} {\text{V}}_{2}$$

where $$1:{ }\,{\text{Hydrofluoric }}\,{\text{acid }}\,{\text{concentration}}$$, $${\text{V}}_{1} :{ }\,{\text{Hydrofluoric}}\,{\text{ acid }}\,{\text{volume}}$$, $${\text{C}}_{2} :{ }\,{\text{Ethanol }}\,{\text{concentration}}.$$, $${\text{V}}_{2} :{ }\,{\text{Ethanol }}\,{\text{volume}}$$.

Following the photo-electrochemical etching process, all prepared PSi substrates underwent a series of tests and experiments to determine the optimal etching parameters, including current densities and etching times^42,44^. X-ray diffraction analysis was conducted using CuKα radiation with a wavelength of 1.54060 Å using XRD diffractometer (XRD6000,Shimadzu) to investigate the structural properties of the deposited films. Morphological parameters were measured using a high-resolution German field emission scanning electron microscope (FESEM) from ZEISS Company. The top layer was examined using an AFM (Atomic Force Microscope) (TT-2 Workshop Compan, USA). The photoluminescence (PL) properties of of the film are investigated using spectrophotometer (PerkinElmer). The Hall effect measurements (Irasol, Hall Effect measurement system, HSR-25AC) were conducted using to estimate the conduction type of GaN. The results showed that the Hall coefficient of the film was positive indicates that the deposited film is p-type.

### Preparation of gallium nitride films

**Figure 2:**
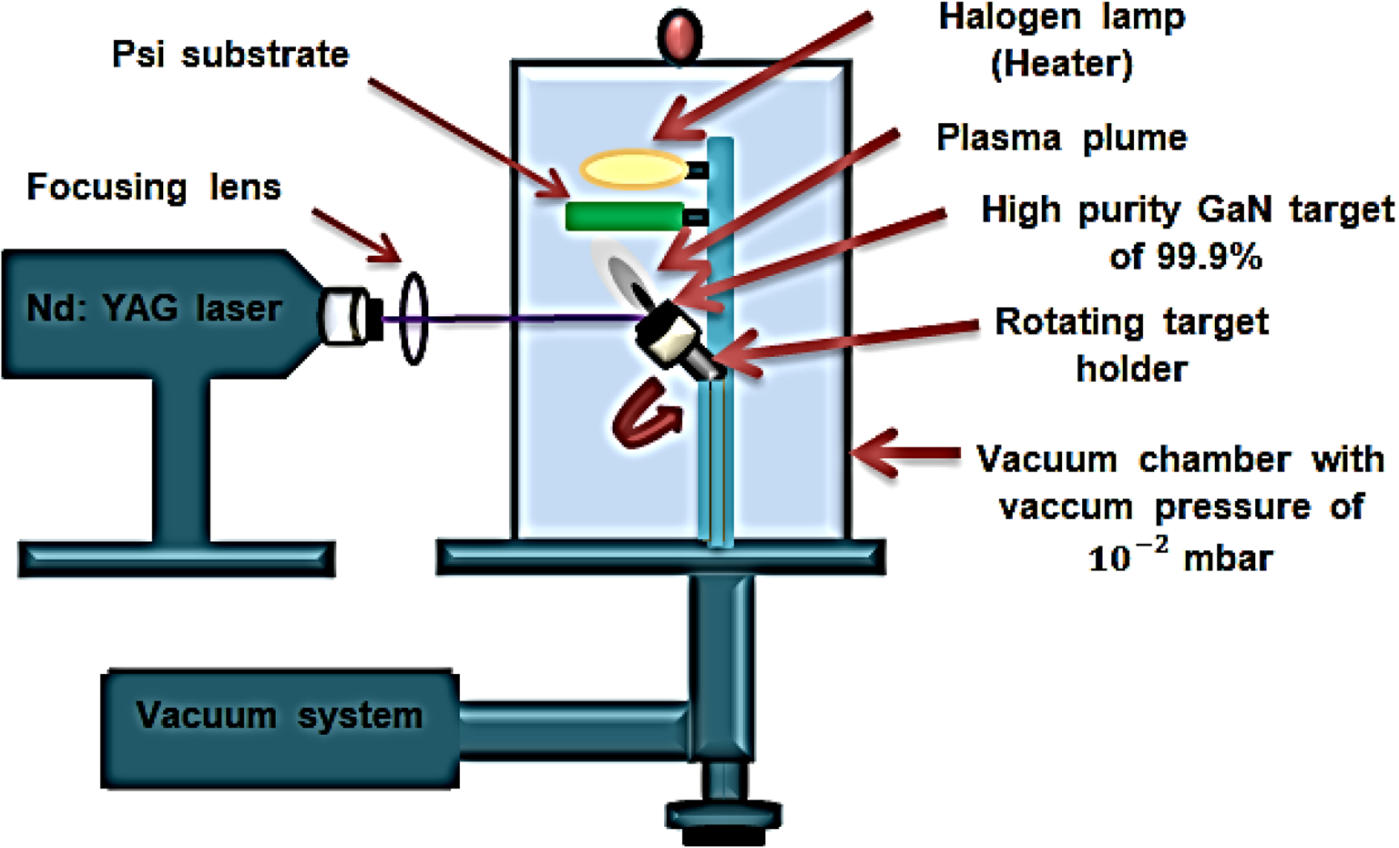
Schematic diagram GaN deposited on PSi substrate using PLD method at different substrate temperatures from 200 to 400 °C^42^.

To deposit GaN films using PLD, a high-purity gallium nitride powder with a 99.9% purity level, supplied by Luoyang Advanced Material Corporation, China, was utilized. The powder was pressed using a hydraulic press at a pressure of 15 kg/cm^2^ to form a circular-shaped GaN target weighing 5 g, with a thickness of 0.5 cm and a diameter of 2 cm. Subsequently, the pressed GaN target was ablated using a Q-switching Nd:YAG laser (RY 280, China) operating at an ambient pressure of 10^–2^ mbar. The laser had an energy of 900 mJ, a wavelength of 355 nm, and a pulse duration of 7 ns, as shown in Table 1. The most favorable outcomes were achieved by depositing GaN material onto the prepared PSi substrate under optimal growth conditions, as illustrated in Fig. 2.

**Table 1:** Growth conditions of Nd: YAG laser deposition system.

Growth conditions	The values
Laser wavelength	355 nm
Pulse energy	900 mJ
Pulse duration	7 ns
Frequency	3 Hz
Repetition rate	300 Hz
Power supply	220 V
Substrate substrate temperature	Psi 200–400 °C

After the pulsed laser deposition procedure, the synthesized PSi layer and the fabricated GaN nanostructure are characterized.

### Electrical properties of GaN/PSi heterojunction

**Figure 3:**
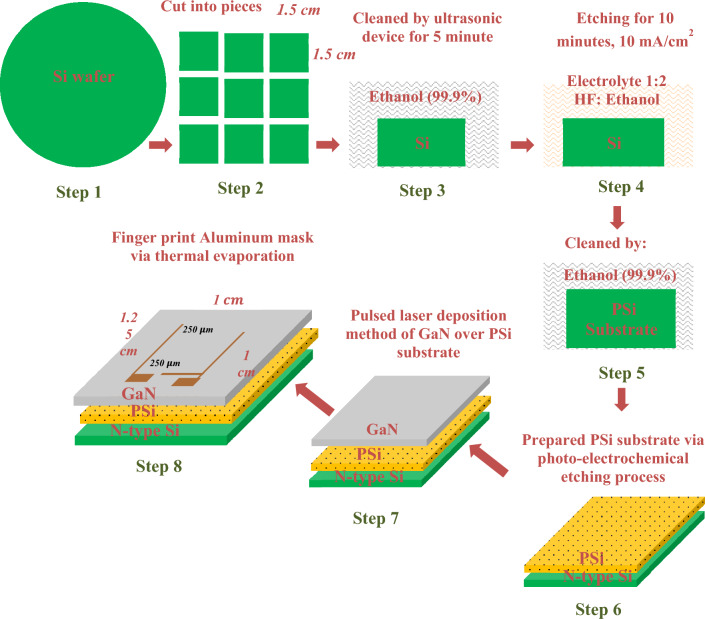
Schematic diagram of preparing GaN/PSi nanostructure using the PLD method.

To establish an ohmic contact, as depicted in Fig. 3, an aluminum mask designed in the shape of a fingerprint was carefully positioned over the fabricated GaN/PSi nanostructure. This mask served the purpose of protecting the nanostructure and preserving its performance. Current–voltage characteristics were measured for both the prepared PSi substrate and the fabricated GaN thin film. This was accomplished using a power supply (Dazheng 30V, 5 A PS-305D from China) and digital multi-meters (UNI-T-UT33C) in the dark, with forward and reverse bias configurations, respectively (TEKR. CDM 250). Additionally, the capacitance–voltage characteristics were studied by using a programmable LCR meter (GW Instek LCR-6100 from Taiwan) in the frequency range of 10 Hz–100 kHz.

## Results and discussion

### Structural properties (XRD)

#### XRD of PSi substrate

**Figure 4:**
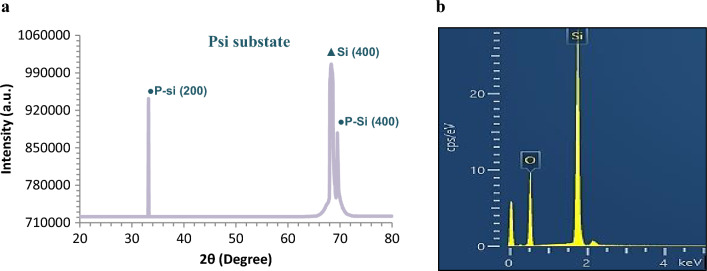
(**a**) XRD patterns of PSi substrate under optimal etching parameters and (**b**) EDX of synthesized PSi substrate under optimum etching parameters.

The XRD patterns of the synthesized PSi substrate, obtained under the optimal working conditions of 10 mA/cm^2^ current density, 10 min etching time, and 24% HF acid concentration, are presented in Fig. 4a. The corresponding analysis of the patterns is provided in Table 2. This figure confirms the presence of XRD peaks associated with both porous silicon and crystalline silicon, which indicates the successful formation of porous silicon. The peaks corresponding to porous silicon are observed at 2θ = 33° and 69°, corresponding to the (200) and (400) crystallographic planes, respectively. As observed, the high intensity peak (400) is splatted into two peaks, one for the Si layer and the other for the PSi layer. As well as, the energy dispersive X-ray (EDX) analysis image confirms existance of O (at 0.5 keV) and Si (at 1.7 keV) as two main peaks to confirm the composition of a produced Psi substrate using aided by a diode laser as shown in Fig. 4b.

**Table 2:** XRD analysis of PSi substrate^42,43^.

Substrate (PSi) orientation (hkl)	2$\uptheta$ (Degree)	$\mathrm{\rm B}$ (Degree)	D (nm)	d (nm)
200	33	0.28	0.27	0.20
400	69.23	0.31	0.13	0.13

#### XRD of GaN/PSi nanostructures

**Figure 5:**
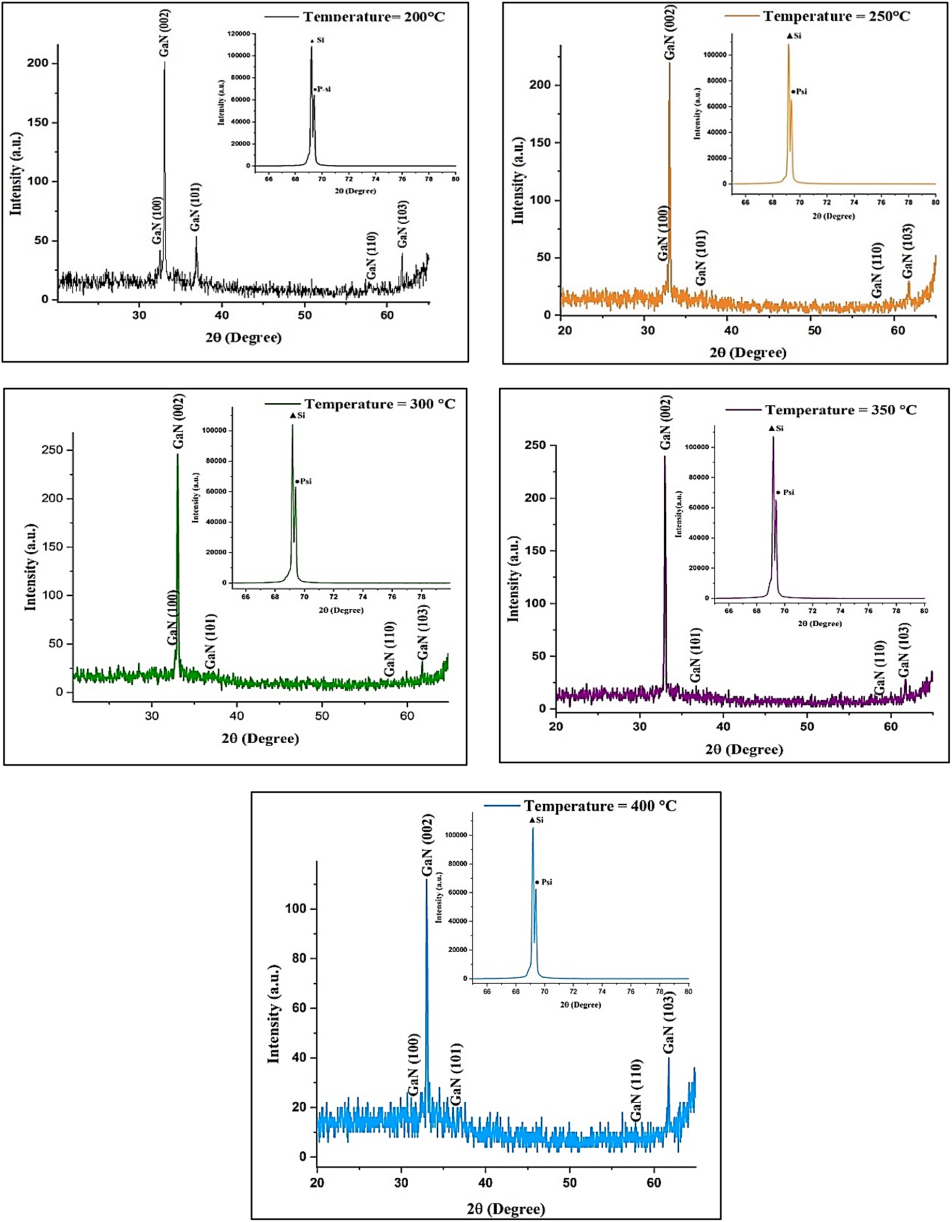
XRD pattern of GaN/PSi nanostructures prepared at various substrate temperatures.

Figure 5 displays the XRD patterns of the GaN/PSi nanostructures fabricated at various substrate temperatures ranging from 200 to 400 °C. The observed peaks along (100), (002), (101), (110), and (103) planes are indexed to GaN (card # 01-074-0243)^52^. The GaN/PSi nanostructures fabricated at 200 °C exhibited a small peak with a narrow full width at half maximum. As the substrate temperatures increased from 200 to 400 °C, both the peak intensity and the full width at half maximum demonstrated gradual changes. Additionally, according to the data presented in Table 3, films deposited at temperatures above 300 °C exhibited a decrease in crystalline quality, attributed to the formation of structural defects.

**Table 3:** XRD analysis of the GaN/PSi nanocrystalline prepared at various substrate temperatures.

Substrate temperature (°C)	GaN films orientation (hkl)	2 $\uptheta$ (Degree)	$\upbeta$(Degree)	D (nm)	d (nm)
200	− 100	32.4	0.18	46.01	0.27
	− 2	33	0.15	55.30	0.27
	− 101	36.84	0.21	39.92	0.24
	− 110	57.7	0.58	15.65	0.15
	− 103	61.7	0.17	54.50	0.15
250	− 100	32.7	0.20	41.44	0.27
	− 2	33	0.13	63.81	0.27
	− 101	36.8	0.42	19.95	0.24
	− 110	57.8	0.24	37.85	0.15
	− 103	61.7	0.23	40.28	0.15
300	− 100	32.76	0.20	41.45	0.27
	− 2	33.04	0.18	46.09	0.27
	− 101	36.64	0.44	19.04	0.24
	− 110	57.72	0.08	113.53	0.15
	− 103	61.76	0.14	66.20	0.15
350	− 2	33	0.18	46.08	0.27
	− 101	36.72	0.18	46.56	0.24
	− 110	57.8	0.16	56.78	0.15
	− 103	61.72	0.22	42.12	0.15
400	− 100	32.48	0.28	29.50	0.27
	− 2	33	0.19	43.66	0.27
	− 101	36.8	0.04	209.57	0.24
	− 110	57.8	0.04	227.15	0.15
	− 103	61.76	0.14	66.20	0.15

The XRD analysis of PSi substrate and GaN film is shown in Tables 2 and 3, respectively. The average crystallite size (D) was calculated using Scherrer's formula^56–58^, as shown in Eq. (2). The interplanar distance (d) was calculated using the formula as shown in Eq. (3)^59–61^.

2 $${\text{D}} = {\text{K}}\uplambda /\upbeta \,\cos \, \uptheta$$

3 $${\text{d}} = {\text{n}}\uplambda /2 \sin \,\uptheta$$

where K = 0.9, $\uplambda$ is the X-ray wavelength, $\upbeta$ is the fullwidth at half maximum, $\uptheta$ is the diffraction angle, and n is a represented positive integer.

As well as, the Williamson-Hall method were used to determined the average crystallite size and strain of fabricated GaN over porous silicon nanostructure as listed in Table 4 and as calculated using Eq. (4)^62,63^.

4 $${\upbeta }_{\mathrm{T}}\mathrm{cos\theta }=\upvarepsilon (4\mathrm{sin\theta })+\frac{\mathrm{K\lambda }}{\mathrm{D}}$$

where $\upbeta$ is total full width at half maximm, $\upvarepsilon$ is strain,$\uptheta$ is diffraction angle, $\mathrm{k}$ is 0.94, $\uplambda$ is the wavelength, and D is thae crystallite size.

**Table 4:** Williamson- Hall analysis of the GaN/PSi nanocrystalline prepared at various substrate temperatures.

Substrate temperature (°C)	$2\uptheta$ (Degree)	${\upbeta }_{\mathrm{T}}$ (Degree)	${\upbeta }_{\mathrm{T}}\mathrm{cos\theta }$	$4\mathrm{sin\theta }$	Average crystallite size (nm)	Strain
200	32.4	0.18	0.0030	1.1159	24.4	0.00765
	33	0.15	0.0025	1.1360		
	36.84	0.21	0.0034	1.2639		
	57.7	0.58	0.0088	1.9300		
	61.7	0.17	0.0025	2.0511		
250	32.7	0.20	0.0033	1.1260	38	1.84E−04
	33	0.13	0.0021	1.1360		
	36.8	0.42	0.0069	1.2625		
	57.8	0.24	0.0036	1.9331		
	61.7	0.23	0.0034	2.0511		
300	32.76	0.20	0.00335	1.1280	17	0.00352
	33.04	0.18	0.00301	1.1374		
	36.64	0.44	0.00729	1.2572		
	57.72	0.08	0.00122	1.9306		
	61.76	0.14	0.0021	2.0529		
350	33	0.18	0.0030	1.1360	47	8.04E−05
	36.72	0.18	0.0029	1.2599		
	57.8	0.16	0.0024	1.9331		
	61.72	0.22	0.0032	2.0517		
400	32.48	0.28	0.0046	1.1186	27	0.00204
	33	0.19	0.0031	1.1360		
	36.8	0.04	0.00064	1.2625		
	57.8	0.04	0.00061	1.9331		
	61.76	0.14	0.0020	2.0529		

### Raman spectroscopy (RS)

#### RS of PSi substrate

**Figure 6:**
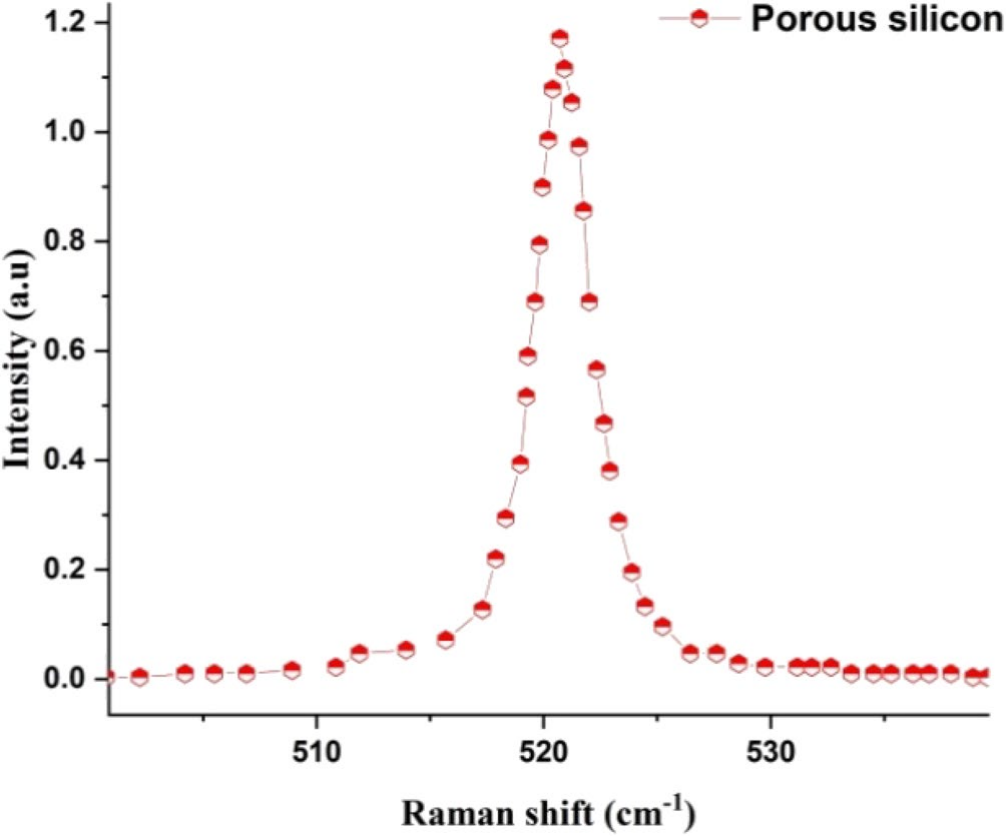
Raman spectra of prepared porous silicon substrate using PECE process assisted by a diode laser under optimum conditions.

Figure 6 dipect the Raman spectrum of prepared PSi substrate by photo-electrochemical etching (PECE). One of the main Raman peaks observed in porous silicon is called “Stokes peak” or the “transverse optical (TO) peak,” which is located around 520–560 cm^−1^. This peak corresponds to the longitudinal optical (LO) phonon mode in crystalline silicon and is shifted to lower frequencies due to the confinement effects in the nanoporous structure^49,64^.

#### RS of GaN/PSi Nano-structures

**Figure 7:**
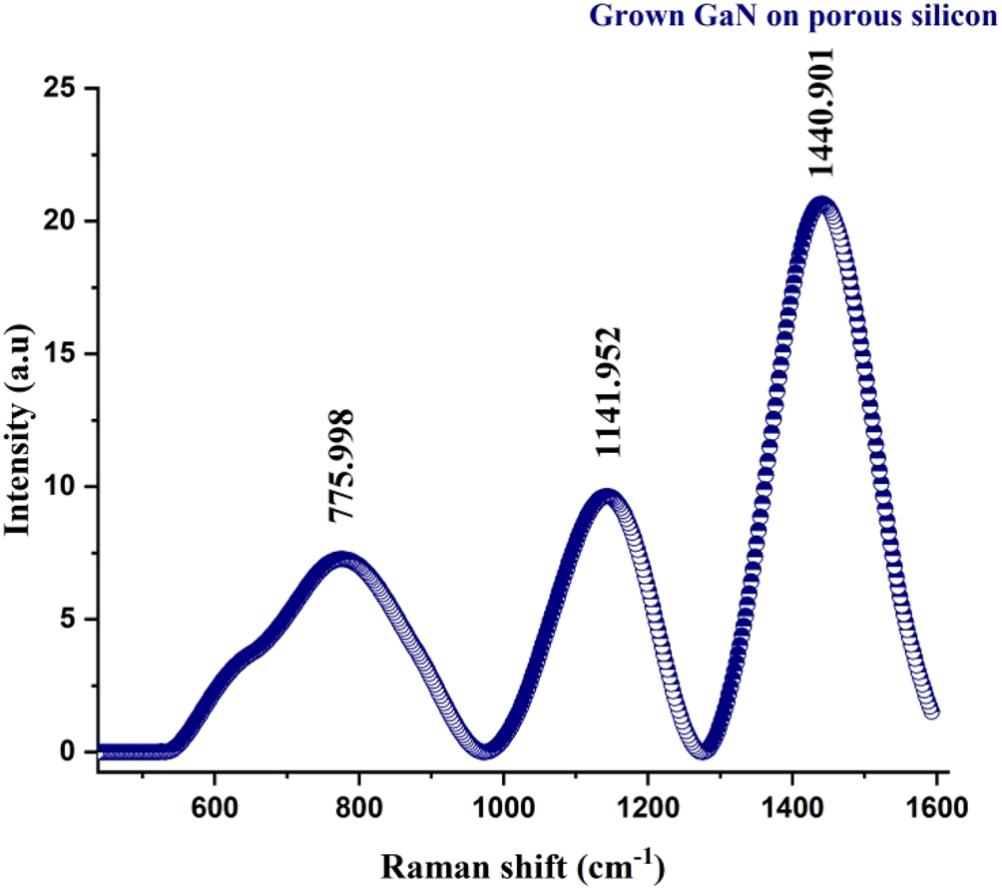
Raman spectrum of prepared GaN on PSi nano-structure using PLD method under 900 mJ laser energy, 355 nm laser wavelength, and optimum substrate temperature of 300 °C.

Figure 7 display Raman spectrum of prepared GaN on PSi nano-structure using PLD method under 900 mJ laser energy, 355 nm laser wavelength, and optimum substrate temperature of 200 °C. Several distinct peaks (775.9, 1141.9, and 1440.9) can be seen in the typical Raman spectra of GaN. The E2 (high) mode is the dominant peak because it is associated with the motion of nitrogen atoms in the crystal lattice. Its location and strength can shed light on the crystal quality and strain of the film they are observing. Additional information about the material can be gleaned from the positions and intensities of other peaks, such as the A1 (TO) and E1 (TO) modes^49,65^.

### Spectroscopic properties

#### PL of PSi substrate

**Figure 8:**
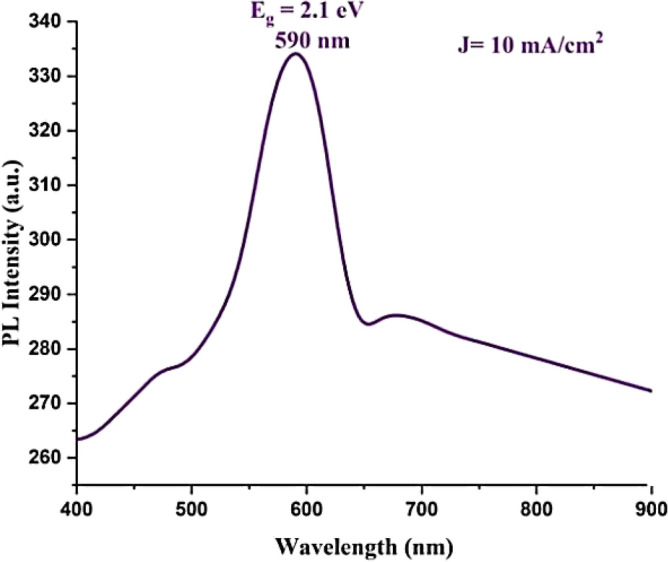
Room temperature PL spectrum of prepared PSi substrate^42^.

The photoluminescence (PL) spectra of the PSi substrate are presented in Fig. 8. PL measurements of the PSi substrate were conducted at room temperature, with an excitation wavelength of 280 nm. Firstly, it is observed that the prepared PSi substrate exhibits a visible yellow band (589 nm) due to surface states and quantum confinement that arise during the photo-electrochemical etching process, as mentioned by Wang^63^. Subsequently, utilizing Eq. (5)^64–66^, the energy band gap of the prepared PSi substrate was determined to be 2.10 eV, which is larger than the energy band gap of silicon (1.11 eV). This difference in energy band gaps can be attributed to the combined effects of quantum confinement and increased surface states, which alter the electronic structure of the material and result in a broader energy gap, as discussed by Canham et al.^67–69^.

5 $${\mathrm{E}}_{\mathrm{gap}}=\frac{\mathrm{hc}}{\uplambda }=\frac{1240}{\lambda (nm)}$$

where ${\mathrm{E}}_{\mathrm{gap}}$ is prepared PSi substrate ‘s energy band gap. h is 6.62 $\times$ 10^–34^ J/s that represent plank’s constant. c is 3 $\times$ 10^8^ m/s that represent light speed. λ is PSi’s wavelength peak (589 nm).

#### PL of GaN/Psi nano-structures

**Figure 9:**
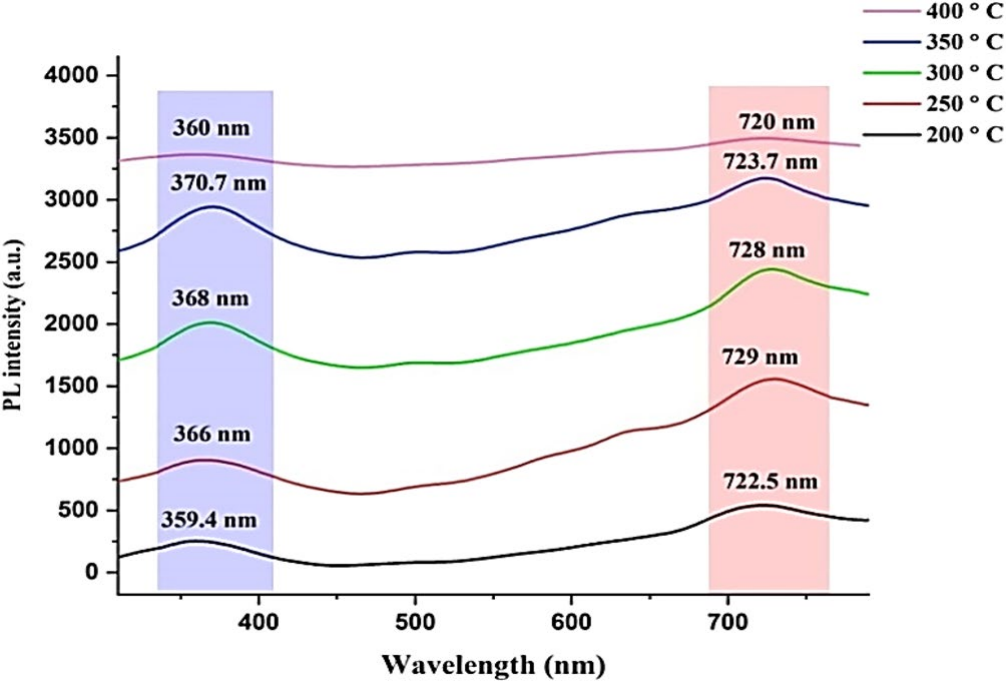
Room temperature PL spectra of GaN/PSi nanostructures prepared at various substrate temperatures.

Figure 9 illustrates the photoluminescence (PL) spectra of GaN nanofilms fabricated at various substrate temperatures ranging from 200 to 400 °C. The PL measurements were conducted on GaN/PSi nanocrystalline films at room temperature, using an excitation wavelength of 320 nm. The results revealed that the GaN nanofilms emitted UV bands corresponding to the characteristic emissions of GaN material, observed at wavelengths of 359.4, 366, 368, 370.7, and 360 nm. Additionally, red bands indicative of the PSi substrate were detected at wavelengths of 722.5, 729, 728, 723.7, and 720.1 nm.

With increasing temperature from 200 to 400 °C, several factors contribute to the observed enhancement in photoluminescence (PL) intensity and reflectance of the GaN film. One of these factors is improved crystal quality, where higher substrate temperatures promote better growth of the GaN film with a more crystalline structure. The elevated temperatures provide greater thermal energy, enabling atoms to move more freely and facilitating the formation of a more orderly crystal lattice. This improved crystal quality results in fewer defects and dislocations, which are known to dampen the photoluminescence (PL) intensity. As a result, the PL intensity of the GaN film increases as the temperature rises. Additionally, the strain is reduced. Temperature influences the lattice matching and strain between the GaN film and the porous silicon substrate. Higher temperatures aid in minimizing the lattice mismatch and strain between the two materials, leading to a lower density of structural defects and dislocations in the GaN film. These defects can function as non-radiative recombination centers, thereby reducing the photoluminescence (PL) intensity. Consequently, by elevating the substrate temperature, the strain is alleviated, resulting in an increase in PL intensity. Furthermore, there is an improvement in surface morphology. The temperature of the substrate also impacts the surface morphology of the GaN film. Higher temperatures during the deposition process facilitate surface diffusion and lead to the smoothing of the film. A smoother surface reduces scattering and enhances light extraction efficiency, resulting in increased reflectance. The enhanced reflectance contributes to higher PL intensity as more emitted photons are reflected back, increasing the likelihood of their detection^70–73^.

### Difused reflected spectroscopy (DRS)

#### DRS of PSi substrate

**Figure 10:**
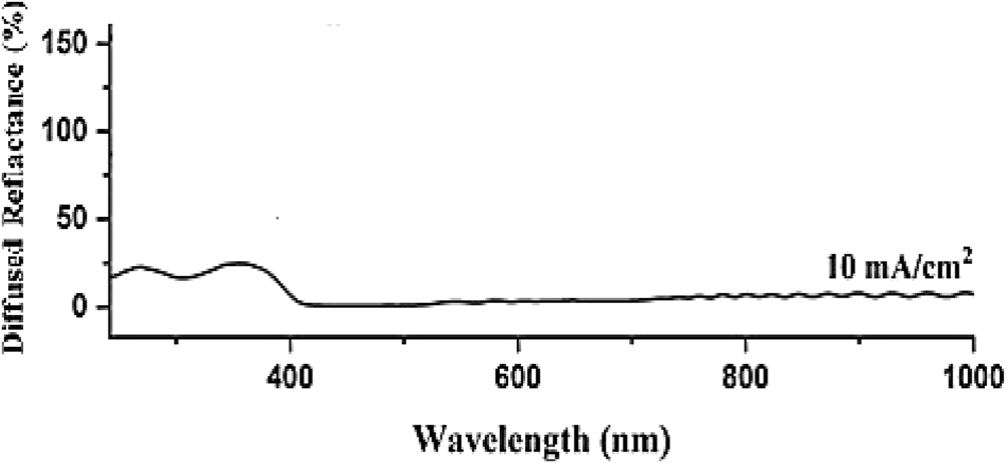
Surface reflectance of PSi substrate.

**Figure 11:**
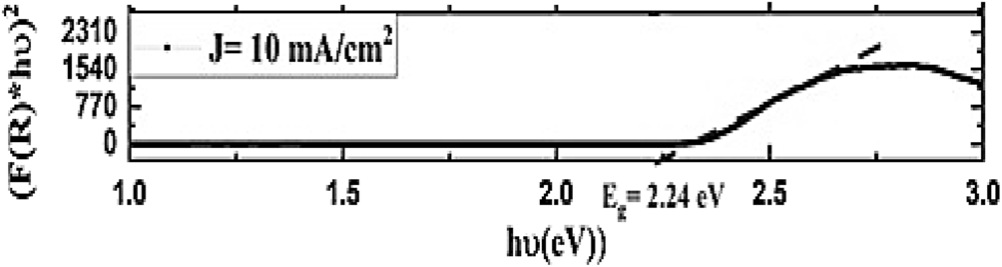
Synthesised PSi substrates Kubelka Munk function.

Diffused reflectance spectroscopy was conducted on the synthesized PSi The measurements were performed in the spectral range from 230 to 1000 nm, as depicted in Fig. 10. Additionally, as shown in Fig. 11, the energy bandgap of the synthesized substrate was determined by calculating the absorption spectra derived from the diffuse reflectance using the Kubelka–Munk function F(R). The calculation was carried out using the following Equation^74–76^:

6 $${\text{F}}_{{\left( {{\text{k}} - {\text{m}}} \right)}} = { }\frac{{\left( {1 - {\text{R}}^{2} } \right)}}{{2{\text{R}}}}$$

where ${\mathrm{F}}_{\mathrm{K}-\mathrm{M}}$ is Kubelka–Munk function, and R is the reflectance obtained from diffused reflected spectroscopy.

#### DRS of GaN/Psi nano-structures

**Figure 12:**
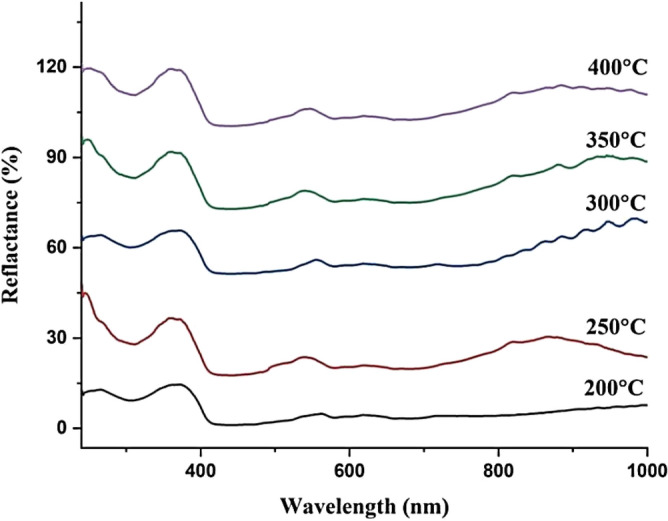
Reflectance of GaN/PSi nanostructures made by the PLD process at substrate temperatures between 200 and 400 °C.

**Figure 13:**
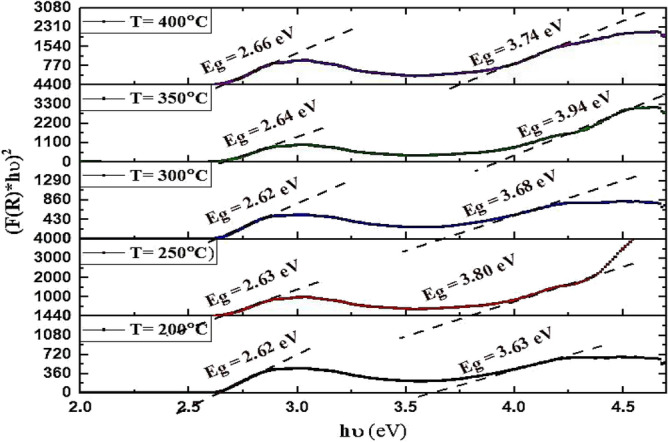
Kubelka–Munk function of GaN/PSi nanostructures made by the PLD process at substrate temperatures between 200 and 400 °C.

The diffused reflectance spectroscopy of the fabricated GaN/PSi nanostructure, prepared using the PLD method at various substrate temperatures ranging from 200 to 400 °C, was measured and is depicted in Fig. 12. Furthermore, Fig. 13 illustrates the energy bandgap, which was calculated from the absorption spectra derived from the diffuse reflectance using the Kubelka–Munk function F(R) with the help of Eq. (5)^74–76^. Notably, at a substrate temperature of 300 °C, the fabricated GaN/PSi nanostructure exhibits significantly higher reflectance in the UV region spectrum compared to other substrate temperatures. As the substrate temperature is increased in the PLD method, several factors contribute to the increased reflectance. One of these factors is improved crystallinity. The elevation of substrate temperature during PLD promotes the growth of a more crystalline GaN film. This higher degree of crystallinity leads to a smoother film surface with fewer defects, resulting in reduced light scattering and increased reflectance. In addition, higher temperatures can enhance the adhesion between the GaN film and the substrate. This improved adhesion minimizes the presence of interfacial gaps and irregularities, thereby leading to improved reflection properties. Moreover, increasing the temperature can also result in a reduction in porosity. Porous silicon substrates typically possess high porosity, which can cause light to scatter and be absorbed within the substrate. By raising the temperature, thermal annealing of the porous silicon occurs, leading to a decrease in porosity. Consequently, more light is reflected off the GaN film rather than being absorbed or scattered within the substrate. Furthermore, elevated temperatures can contribute to a reduction in surface roughness. This is achieved through surface diffusion processes promoted by the increased temperature, resulting in the smoothening of the GaN film surface. A smoother surface reduces light scattering and enhances reflectance^37,77,78^.

### Surface topography AFM

#### AFM of PSi substrate

**Figure 14:**
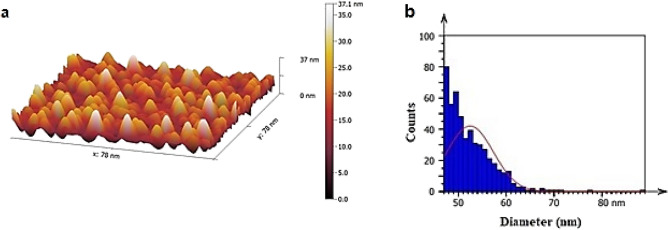
(**a**) AFM image of prepared PSi substrate; (**b**) grain size distribution image of prepared PSi substrate^42,43^.

Figures 14a,b present an AFM image and the distribution of grain sizes, respectively, for a prepared PSi substrate that underwent laser-assisted photo-electrochemical etching to investigate surface topography. Upon etching for 10 min, uniformly distributed pores formed across the entire surface, exhibiting an elongated oval shape. The AFM parameters of the prepared PSi substrate are provided in Table 5. Notably, nanometer-scale analysis was conducted to determine the particle size distribution of the PSi substrate after preparation.

**Table 5:** AFM parameters of synthesized PSi substrate^42,43^.

Root-mean-square height (nm)	Maximum height (nm)	Average surface roughness (nm)	Average diameter (nm)
11.88	37	9.40	52.46

#### AFM of GaN/PSi Nano-structures

**Figure 15:**
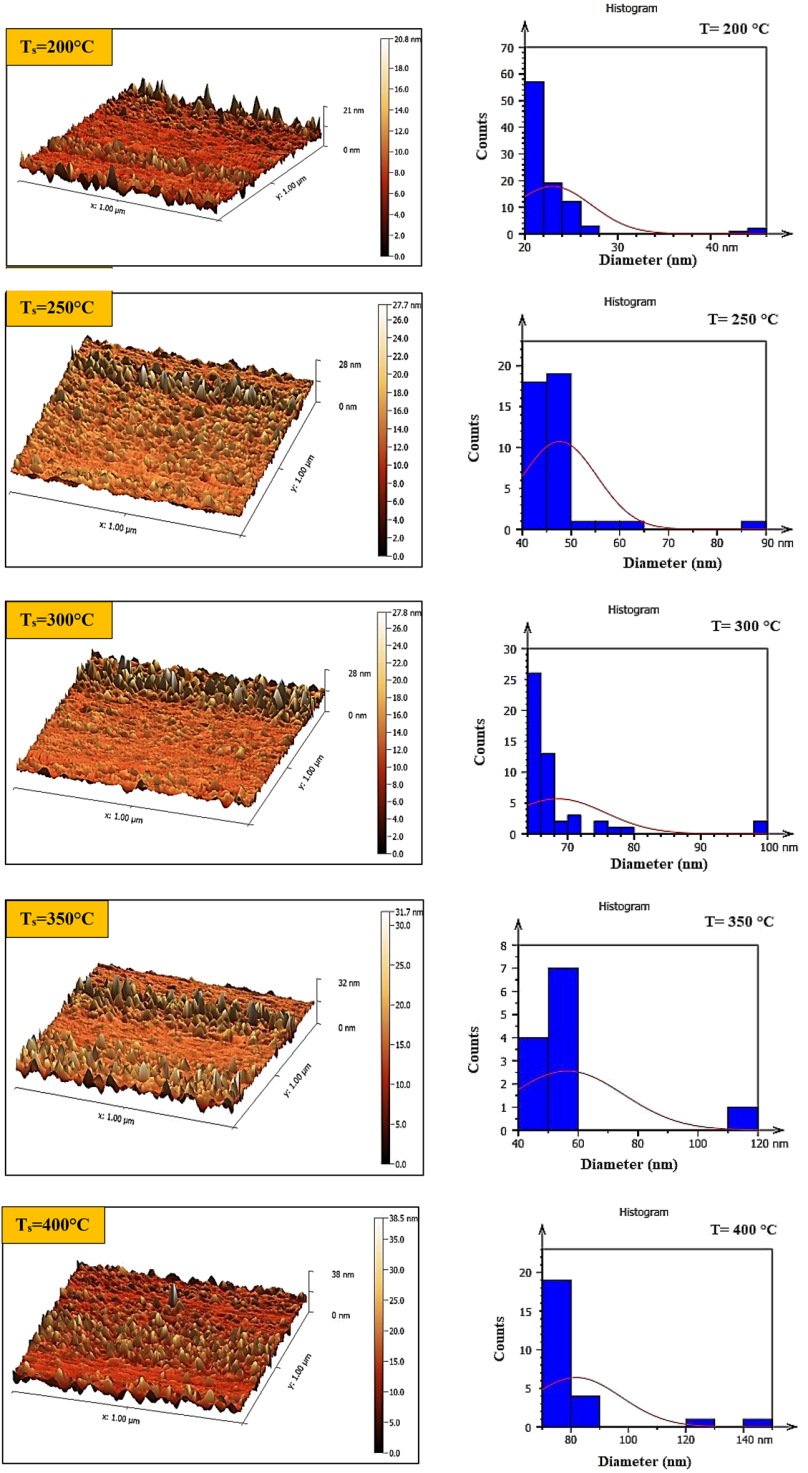
AFM and grain size distribution images of GaN/PSi nanostructures prepared at various substrate temperatures.

Figure 15 shows three-dimensional AFM images and the size distribution of the grains in GaN nanofilms fabricated at different substrate temperatures. As shown in Table 6, increasing the substrate temperature from 200 to 400 °C resulted in smoother surfaces and reduced surface roughness, with the lowest roughness observed at a substrate temperature of 400 °C. The researchers attributed the improvement in surface morphology to the increased mobility of the GaN adatoms at higher substrate temperatures^79,80^.

**Table 6:** AFM parameters of prepared GaN/PSi nanostructures using PLD process at different substrate temperatures 200 to 400 °C.

${\mathrm{T}}_{\mathrm{s}}$ (°C)	Root-mean-square height (nm)	Average surface roughness (nm)	Average diameter (nm)
200	9.38	35.48	22.95
250	19.19	28.06	47.68
300	39.04	20.15	56.31
350	24.17	14.36	68.56
400	33.74	7.69	81.82

### Surface morphology FESEM

#### FESEM of PSi substrate

**Figure 16:**
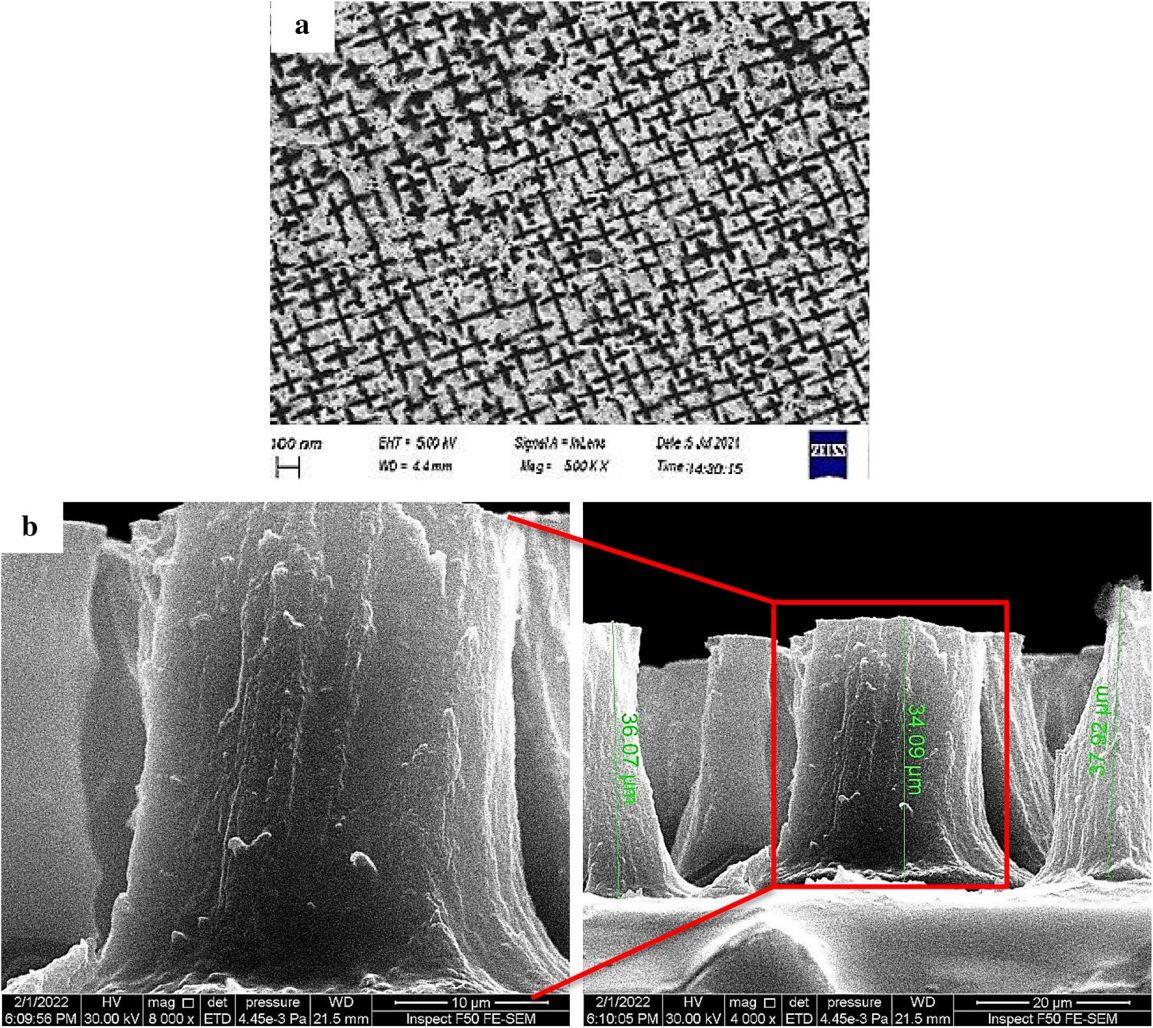
FE-SEM images of synthesized PSi substrate, (**a**) surface area image, and (**b**) cross -section image^44^.

Figures 16a,b display FE-SEM images of the surface and cross-section, respectively, of the PSi substrates, providing insight into the surface morphology.

Consistent with the findings of Omar et al.^51^, the surface pores exhibit a star-like appearance and maintain a consistent elongated shape across the entire surface. This characteristic appearance is attributed to the utilization of low-resistance n-type silicon (100) during the preparation process. Additionally, the thickness of the PSi layer was determined to be 36.02 nm based on a cross-sectional FE-SEM image^43,81^.

#### FESEM of GaN/PSi nano-structures

**Figure 17:**
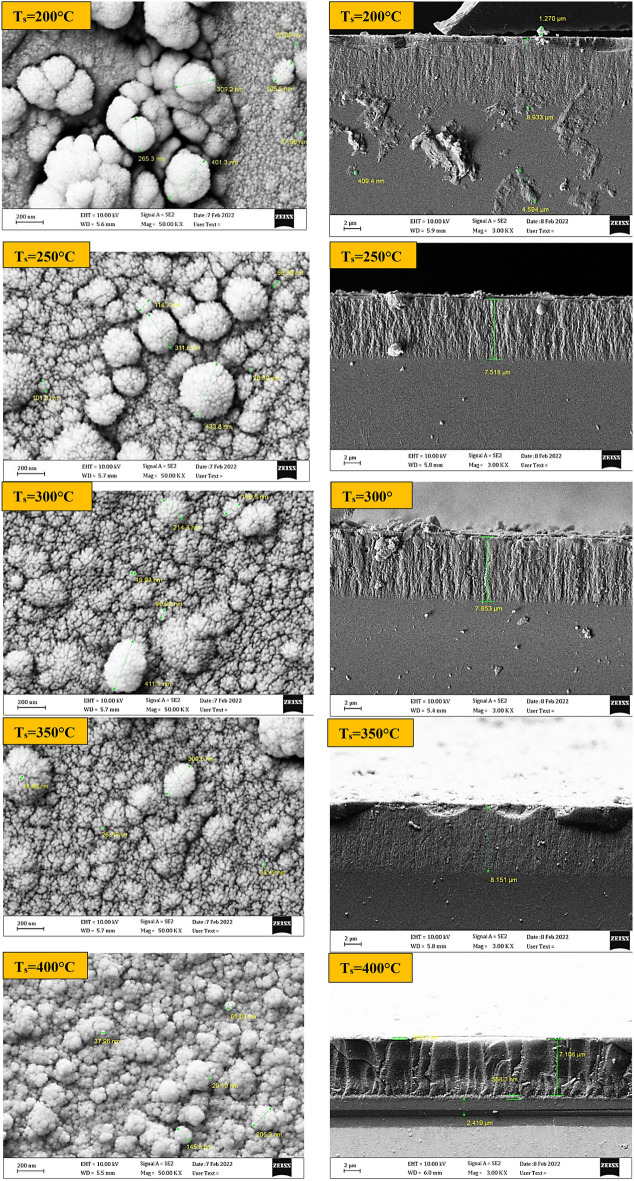
FE-SEM and cross-section images of GaN/PSi nanostructures made by the PLD process at substrate temperatures between 200 and 400 °C.

Figure 17 depicts FE-SEM images of GaN/PSi nanostructures fabricated using the PLD method, highlighting the influence of varying substrate temperatures ranging from 200 to 400. Microscopy and nanotechnology techniques were utilized to examine the surface morphology of the GaN films. The resulting GaN nanoparticles completely covered the PSi substrate, forming spherical particles of uniform and homogeneous size, resembling the shape of cauliflower. Additionally, the cross-section of the GaN nanofilms was examined using FE-SEM to determine their thickness as listed in Table 7. It was observed that the substrate temperature had a significant impact on the surface morphology of the GaN thin films grown through PLD. At low substrate temperatures of 200 and 250 °C, the GaN thin films demonstrate poor crystallinity, a high defect density, and a rough surface morphology, resulting in FE-SEM images with low resolution and contrast. Conversely, at high substrate temperatures of 300, 350, and 400 °C, the GaN thin films exhibit improved crystallinity, a lower defect density, and a smoother surface morphology, leading to higher resolution and contrast in the FE-SEM images. The fabricated GaN/PSi nanostructures have a thickness of 2.426 μm.

**Table 7:** The thickness of GaN films deposited on PSi substrate at laser energy of 900 mJ as a function of substrate temperature.

Substrate temperature (°C)	Thickness (μm)
200	3.8
250	3.5
300	2.42
350	2.3
400	2.2

Table 7 displays the thickness of the created GaN on PSi nanostructures at various temperatures, and it can be shown that the nanostructure has a thickness of 2.426 m when it is fabricated at 300 °C. The average crystallite size, calculated using the Sherrer Eq. (2), decreases with decreasing film thickness in GaN on Psi nanostructures fabricated via the PLD process at substrate temperatures ranging from 200 to 400 °C. This trend can be attributed to several factors. One explanation is the increased likelihood of defects during thin film formation, such as dislocations and stacking faults. Smaller crystallites can develop at these flaws, which serve as nucleation sites. Moreover, strain and stress inside the thin film can also affect the size of the crystallites. Larger crystallites may break up into smaller ones as strain and stress levels rise due to reduced film thickness as referred in Tables 3 and 7.

### Capabilities in electricity

#### Psi substrate’s electrical characteristics

**Figure 18:**
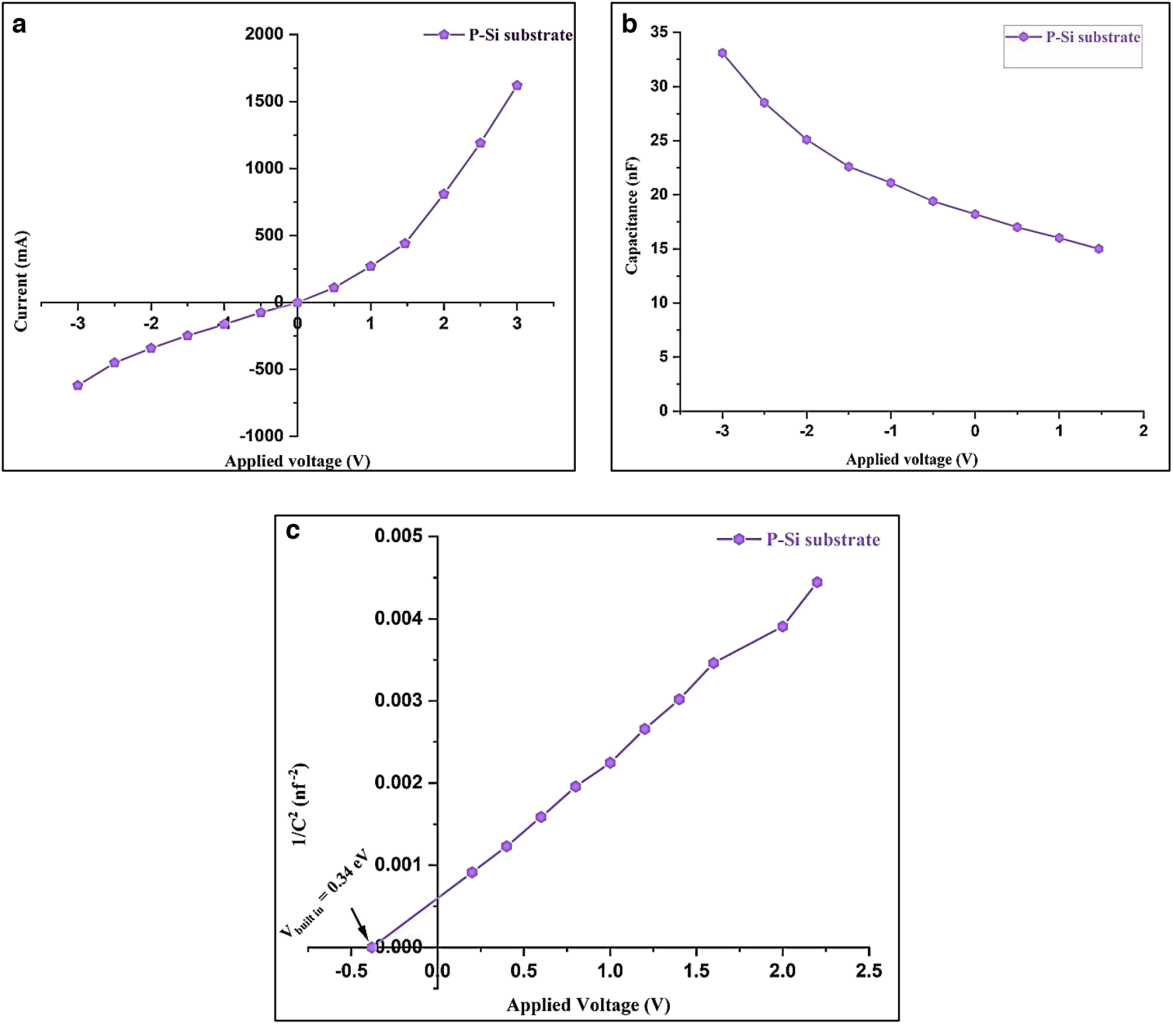
(**a**) Dark I–V characteristics at forward and reverse, (**b**) C–V characteristics, and (**c**) 1/C^2^ versus voltage^42,43^.

The current–voltage, capacitance–voltage, 1/C^2^ and voltage characteristics of the photodetector are shown in Fig. 18a–c, respectively.

As shown in Fig. 18a, a depletion region between the PSi and c-silicon resulting in a rectifying characteristic^82,83^. As the voltage is applied, the current flowing through the PSi substrate increases due to charge transfer. On the other hand, Fig. 18b demonstrates a decrease in capacitance as the bias voltage increases from 0 to 3 V. This finding can be attributed to the expanding depletion region that occurs with increasing bias voltage, subsequently leading to a decrease in capacitance^84,85^.The relationship between 1/C^2^ and voltage on a PSi substrate after fabrication is seen in Fig. 18c. The built-in potential was determined by extrapolating the linear segment of the curve to a 1/C^2^ value of 0 points. The calculated value of the built-in potential was found to be 0.34 V.

#### Electrical properties of GaN/PSi nano-structures

**Figure 19:**
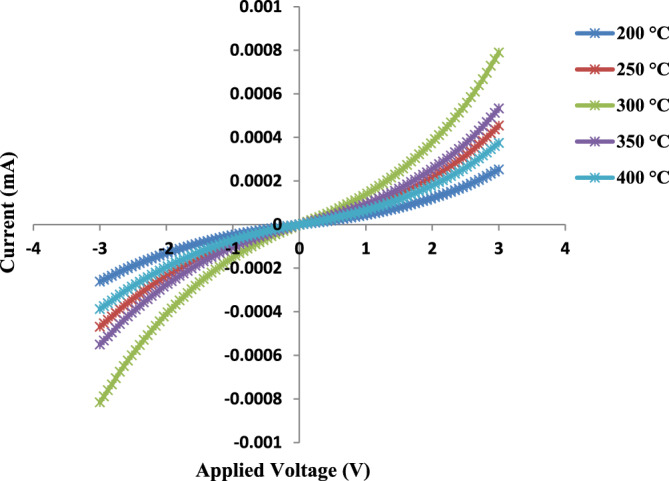
PLD-fabricated GaN/PSi nanostructure dark I–V properties at substrate temperatures between 200 and 400 °C under forward and reverse biases.

Figure 19 illustrates the dark I–V characteristics of GaN/PSi nanostructures fabricated at different substrate temperatures. It is observed that increasing the substrate temperatures from 200 to 300 °C results in improved crystallinity, reduced defect density, and increased carrier mobility. This can be attributed to the higher substrate temperature promoting better surface diffusion and increased mobility of adatoms, leading to the formation of high-quality crystalline structures. Furthermore, higher substrate temperatures can enhance the stoichiometry of the deposited films and reduce impurity incorporation. However, above 300 ℃, the material properties begin to degrade due to thermal damage^86,87^.

**Figure 20:**
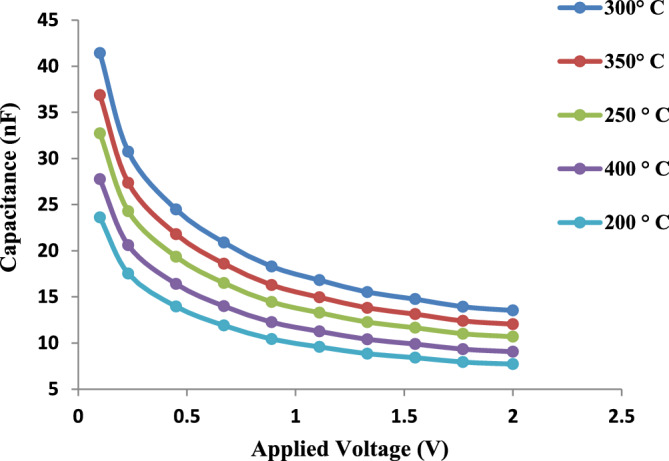
C–V characteristics of GaN/PSi nanostructures made by the PLD process at substrate temperatures between 200 and 400 °C.

Figure 20 depicts the Capacitance vs voltage characteristics of GaN/PSi nanostructures fabricated using the PLD process at substrate temperatures ranging from 200 to 400 °C under an applied voltage of 0–2 V. The increase in capacitance observed at a substrate temperature of 300 °C can be attributed to an increase in the doping concentration and a decrease in the defect density. However, at temperatures above 300 °C, the capacitance decreases due to the formation of Ga clusters, which reduce the effective doping concentration^40,88^.

**Figure 21:**
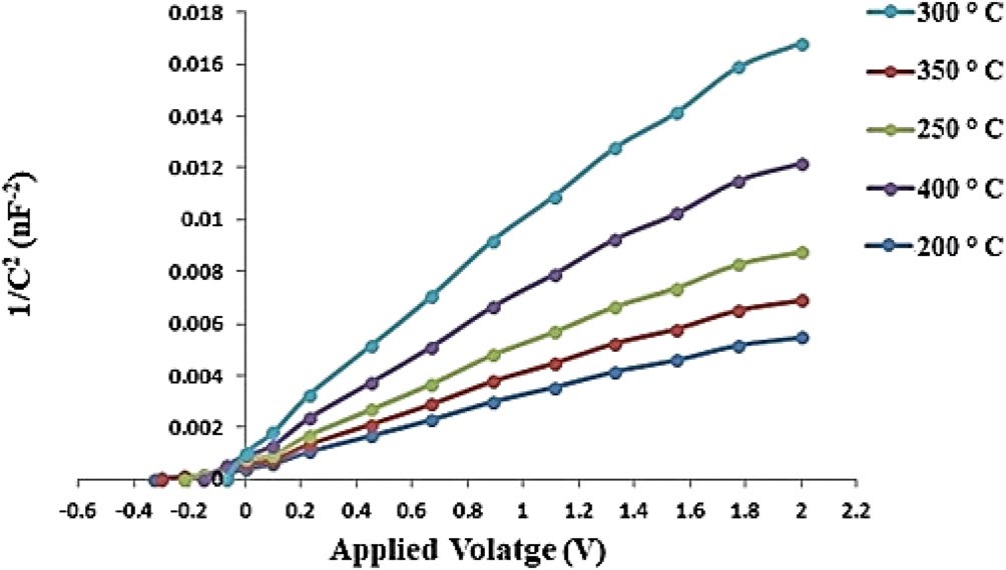
1/C^2^–V characteristics of GaN/PSi nanostructures made by the PLD process at substrate temperatures between 200 and 400 °C.

Figure 21 illustrates the 1/C^2^ versus voltage characteristics of GaN/PSi nanostructures fabricated using the PLD process on different substrates. The 1/C^2^ versus V characteristic is a plot of the inverse square of the capacitance, commonly used to analyze the interface properties of semiconductor devices. The built-in potential was found to be 0.32, 0.30, 0.21, 0.16, and 0.14 V at substrate temperatures of 200, 250, 300, 350, and 400 °C, respectively. It is observed that the built-in voltage of the GaN/PSi nanostructures decreases from 0.32 to 0.14 V, indicating a decrease in the defect density within the GaN/PSi nanostructure^89,90^.

### Effect of substrate temperatures on the performance properties of GaN/Psi heterojunction photodiode

**Figure 22:**
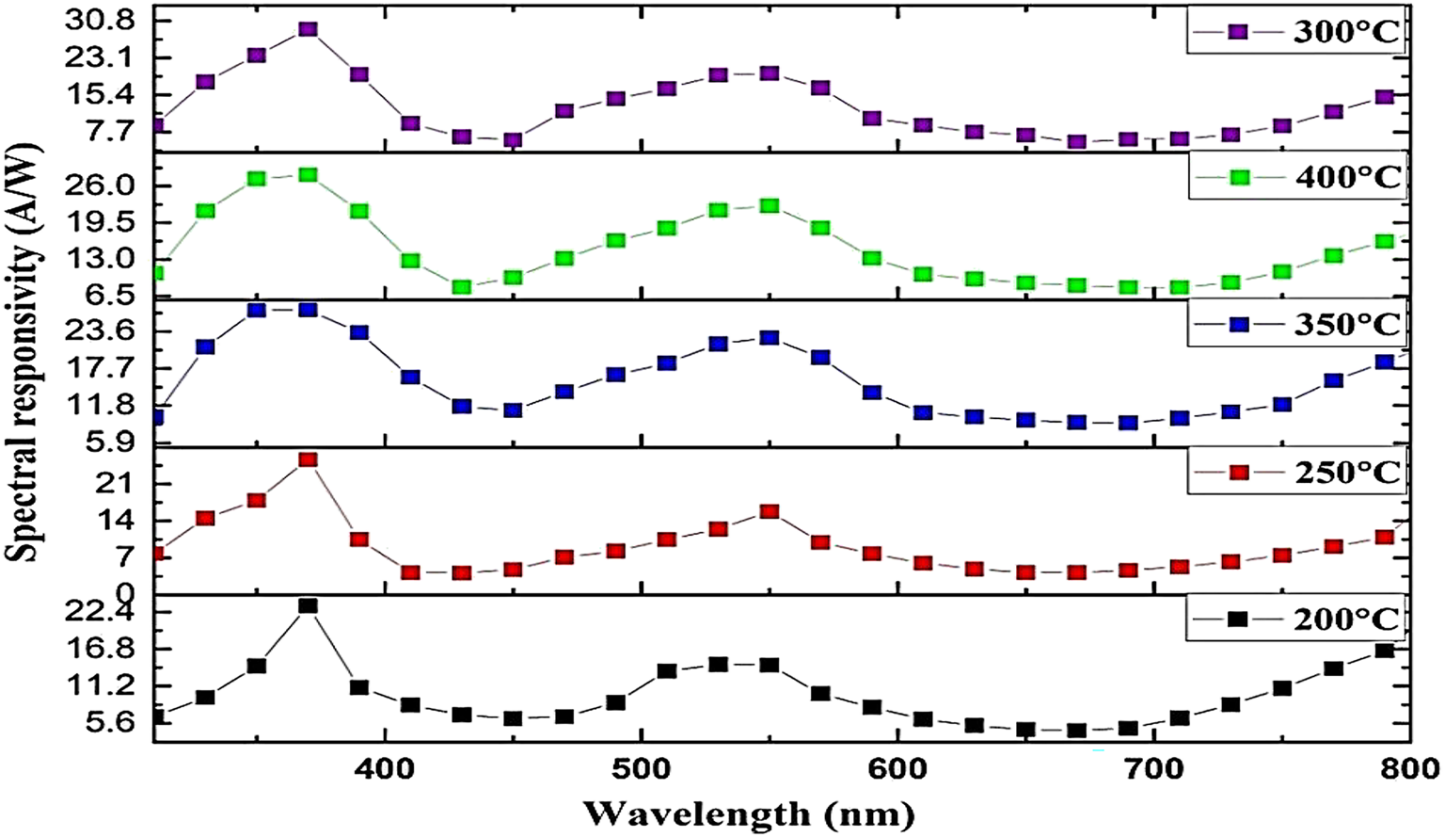
Spectral responsivity of GaN/ PSi heterojunction photodetectors fabricated at various substrate temperatures.

**Figure 23:**
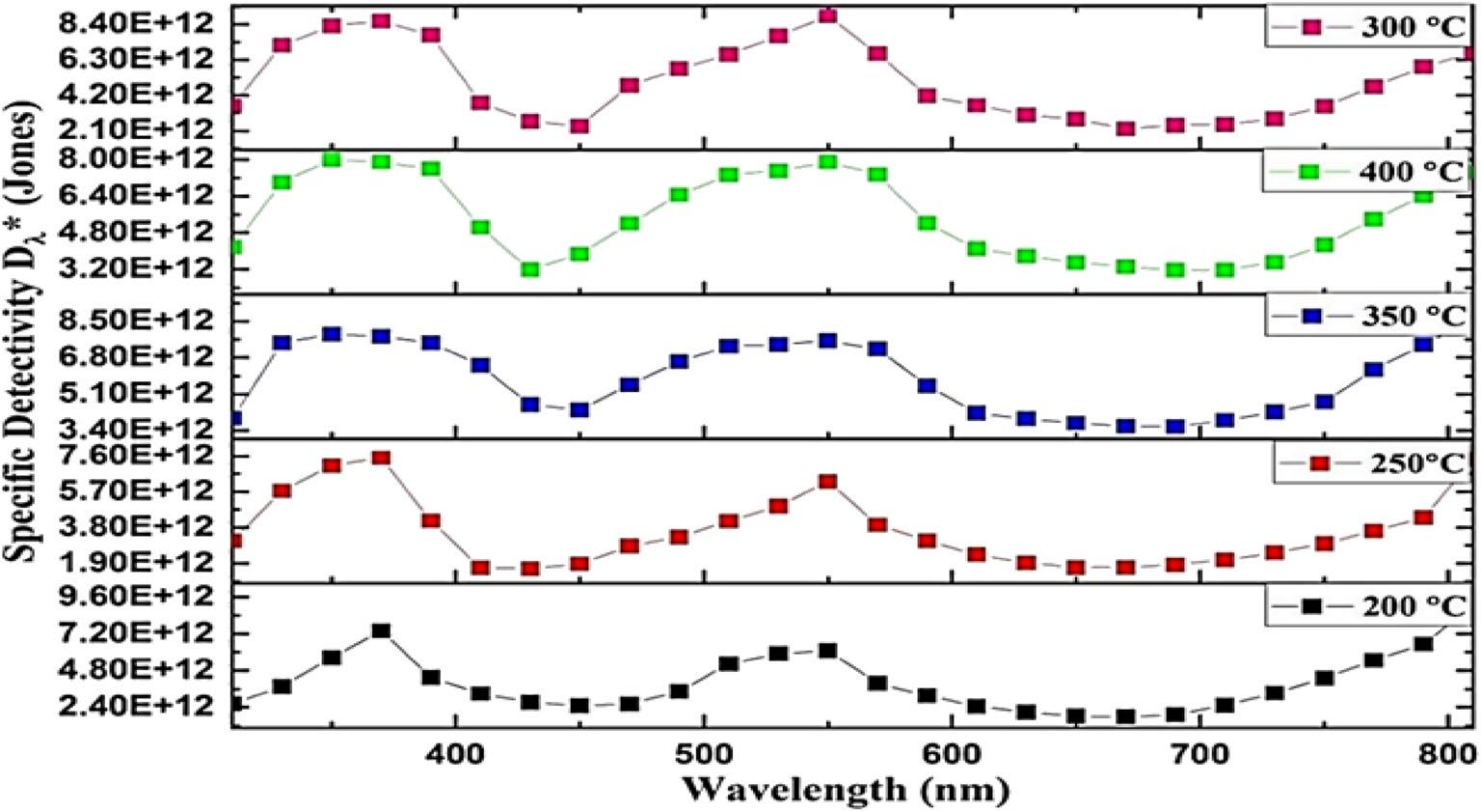
Specific detectivity of GaN/ PSi nanostructures made by the PLD process at substrate temperatures between 200 and 400 °C.

**Figure 24:**
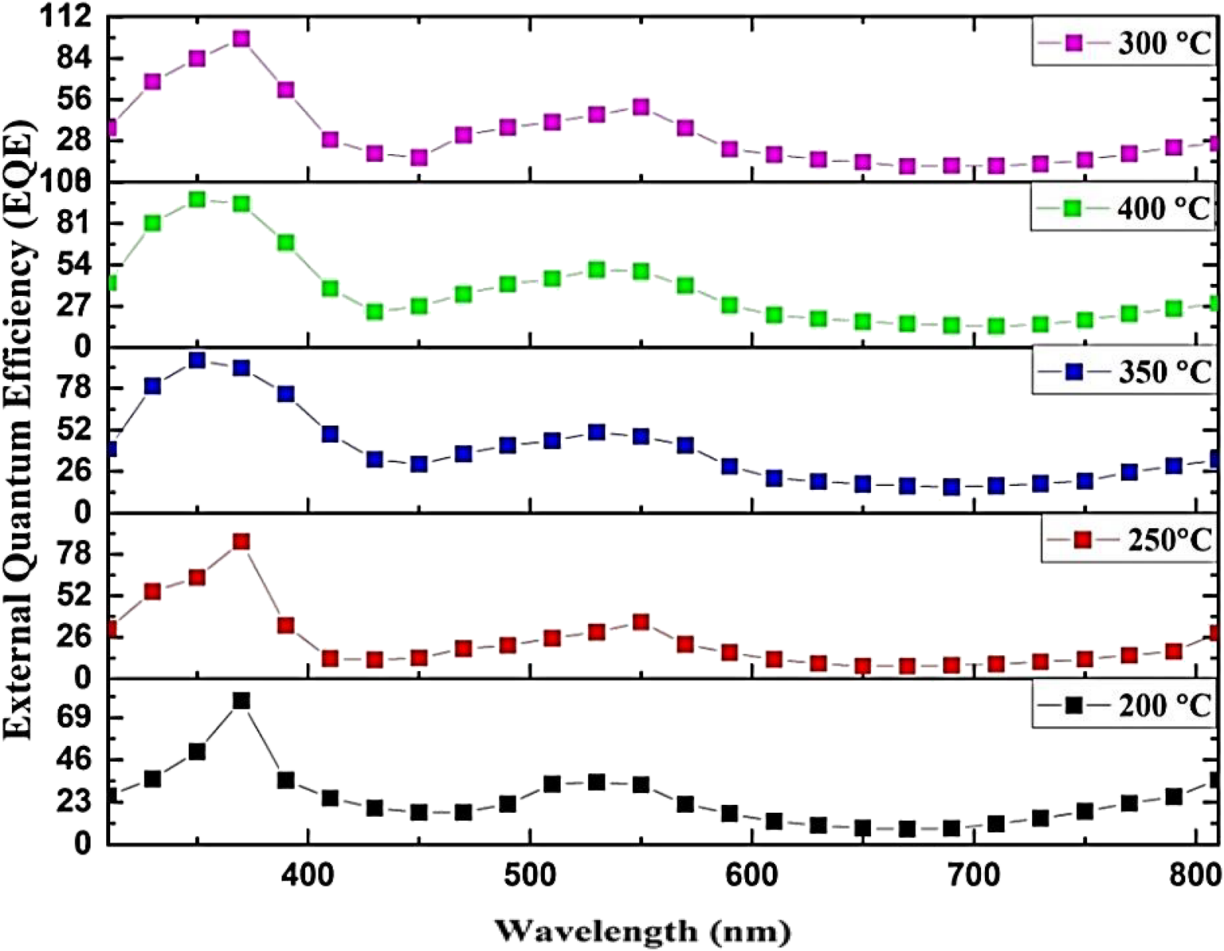
Impact of substrate temperature on the EQE of the photodetectors.

The performance properties, including responsivity (R_*λ*_*), specific detectivity (D*_λ_), and external quantum efficiency (EQE), of the fabricated GaN/PSi heterojunction using the PLD method with optimal laser parameters (900 mJ laser energy, 355 nm laser wavelength) at different substrate temperatures, were determined and are depicted in Figs. 22, 23, and 24. The equations representing R_λ_*, D*_*λ*_, and EQE are given as Eqs. (6), (7), and (8), respectively^91,92^.

7 $${\text{R}}_{{\uplambda }} = \frac{{{\text{I}}_{{{\text{ph}}}} }}{{\text{P}}}$$

where I_ph_ is the photocurrent (Ampere), and P is the incident power (Watt). The specific detectivity D* is given by^93,94^:

8 $${ }({\text{D}}^{*}_{{\uplambda }} ) = \frac{{{\text{R}}_{{\uplambda }} \sqrt {\text{A}} }}{{\sqrt {2{\text{qI}}_{{\text{d}}} } }}$$

where A is the area of photodetector, ${\mathrm{I}}_{\mathrm{d}}$ is the dark current of photodetector, and q is the electron charge. The eternal quantum efficiency can be given by^95,96^:

9 $$\left( {{\text{EQE}}} \right) = \frac{{1240{\text{ R}}_{{\uplambda }} }}{{\lambda_{nm} }}$$

The wavelength of the incident photon is denoted by $\uplambda$, while the spectral responsivity is denoted by ${\mathrm{R}}_{\uplambda }$.

Figure 22 illustrates the measured spectral responsivity of fabricated GaN/PSi heterojunction photodiode using the PLD process at different substrate temperatures from 200 to 400 °C. It’s observed that the responsivity increased with increasing substrate temperature up to 300 °C and then decreased at higher temperatures of 350 and 400 °C. They attributed this behaviour to increased crystalline quality and reduced defect density of the fabricated GaN/PSi nano-structures photodiode at higher substrate temperatures (350 and 400 °C)^97,98^. The high responsivity of the fabricated photodetectors is due to the presence of two heterojunctions: GaN/PSi and PSi/C-Si. Two response peaks are found at 380 and 540 nm, with the first one being attributed to the absorption edge of the GaN film, while the second peak belongs to the porous silicon substrate. The maximum responsivity was 29.03 A/W at 380 nm and 19.86 A/W at 540 nm for the photodetector prepared at a substrate temperature of 300 °C. We believe that the reason behind this finding is the large depletion width that extends toward the film surface, enhancing the responsivity of short wavelengths. When the film thickness is reduced, it results in an increased transmission of photons to the silicon substrate, especially for those with wavelengths (λ) greater than the GaN cut-off wavelength (λcut-off). This phenomenon leads to photon absorption in the porous silicon substrate, thereby enhancing responsivity in the visible region^99,100^.

In addition, Fig. 23 illustrates the specific detectivity of the fabricated GaN/PSi nanostructures. It is observed that the density of defects decreases as the substrate temperature increases. Consequently, a substrate temperature of 300 °C is determined to be the optimal condition during the PLD growth process to achieve high-quality GaN/PSi nanostructures.

The computed EQE of a PLD-process-fabricated GaN/PSi heterojunction photodiode at substrate temperatures ranging from 200 to 400 °C is shown in Fig. 24. It’s observed the EQE increased with increasing substrate temperature from 200 to 300 °C, and then decreased at higher temperatures of 350 and 400 °C. They attributed this behaviour to improving crystal quality at higher substrate temperatures, which reduced the non-radiative recombination centres and enhanced the radiative recombination^101,102^.

**Figure 25:**
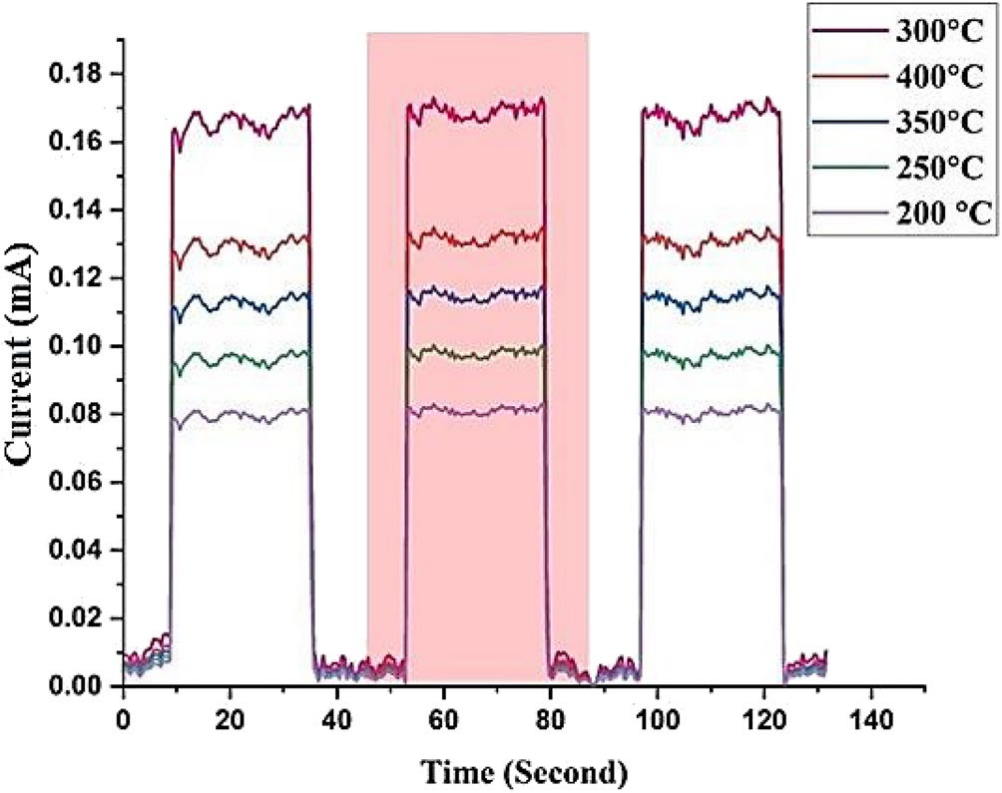
A switching characteristic of fabricated GaN/ PSi heterojunction photodiode at different substrate temperatures.

**Figure 26:**
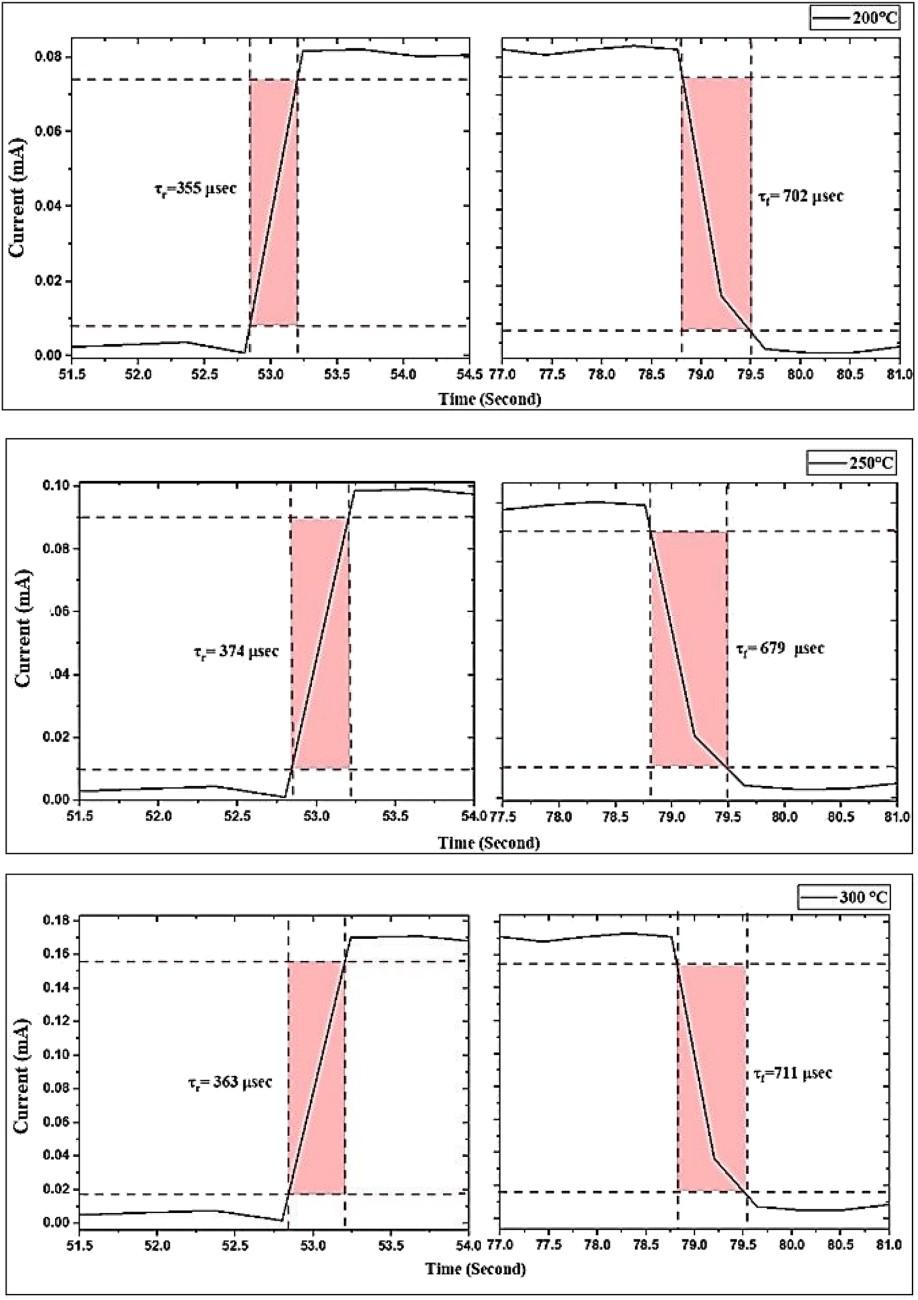

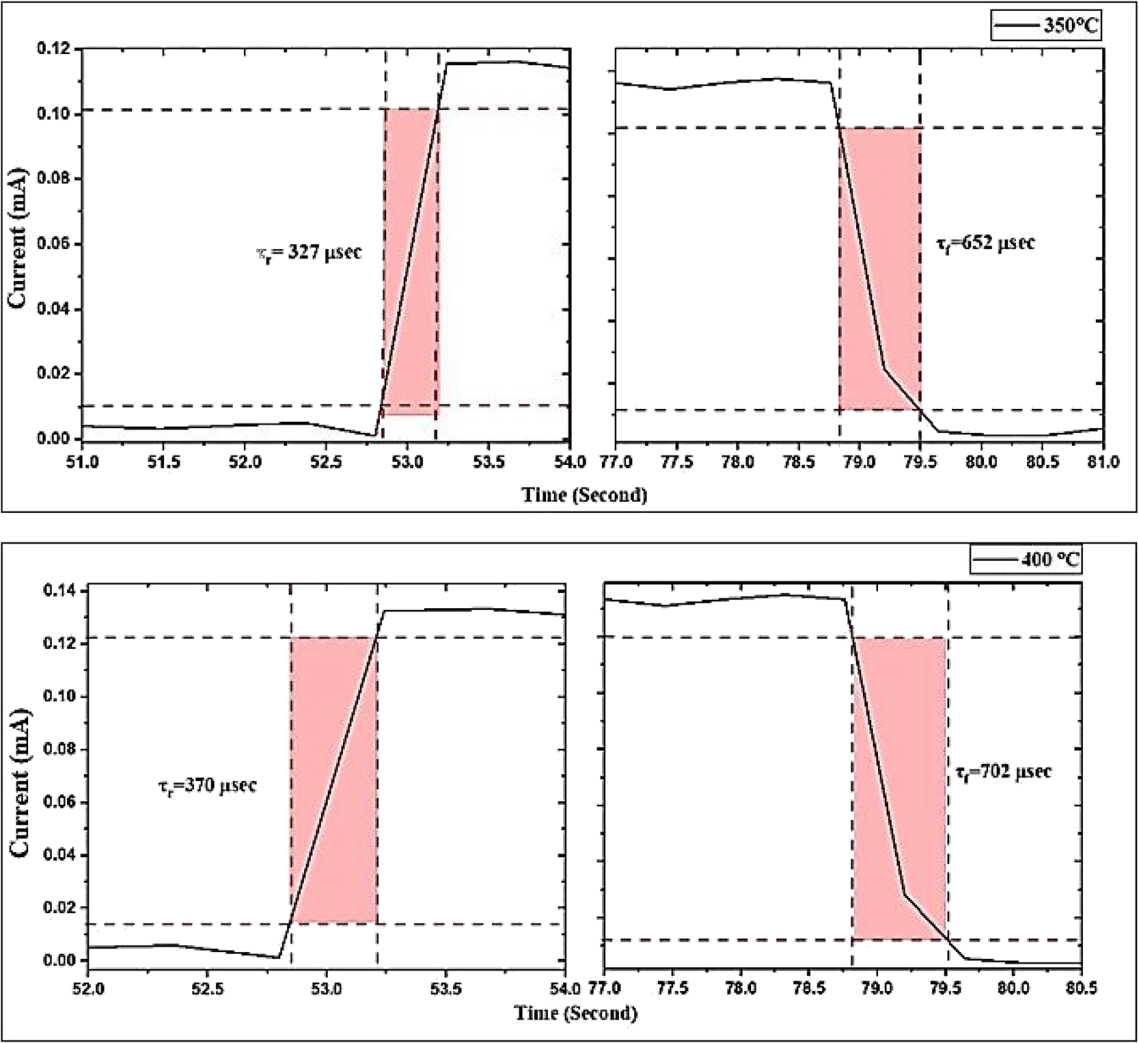
Rise time and fall time of GaN/ PSi nanostructures made by the PLD process at substrate temperatures between 200 and 400 °C.

All of the GaN/PSi heterojunction photodetectors listed in Table 8 were measured at 100 mW/cm^2^ using a Keithley 2400, and their switching characteristics are presented in Figs. 25 and 26 for substrate temperatures ranging from 200 to 400 °C. The switching tests consisted of three separate cycles, each lasting 25 s on and 18 s off. The rise time of the photodiodes was measured from 10 to 90% of the peak signal, while the fall time was measured from 90 to 10% of the peak signal, as depicted in Fig. 26. It is observed that increasing the substrate temperature from 200 to 300 °C during the PLD growth process of GaN thin films results in a reduction in the rise time and fall time of electronic devices based on GaN, owing to the improvement in crystalline quality. Specifically, at 300 °C, the fabricated GaN/PSi nanostructure photodiode exhibited a rise time of 328 μs and a fall time of 617 μs.

**Table 8:** Figures of merit of GaN/PSi nanostructures photodetector fabricated at various substrate temperatures.

${\mathrm{T}}_{\mathrm{s}}$ ($\mathrm{^\circ{\rm C} }$)	${\mathrm{R}}_{\uplambda }$ (A/W)	${{\mathrm{D}}^{*}}_{\uplambda }$×10^12^ (Jones)	EQE (%)	${\uptau }_{\mathrm{r}}$ ($\mathrm{\mu s}$)	${\uptau }_{\mathrm{f}}$ ($\mathrm{\mu s}$)
200	23.36 at 370 nm	7.4	78.28	355	702
	14.46 at 550 nm	6.1	33.8		
250	25.62 at 370 nm	7.5	85.88	374	679
	15.78 at 555 nm	6.2	35.5		
300	29.03 at 370 nm	8.6	97.2	363	711
	19.86 at 575 nm	8.9	50.89		
350	27.05 at 370 nm	7.8	90.6	327	652
	22.57 at 555 nm	7.6	47.9		
400	28.02 at 370 nm	7.9	93.9	370	702
	22.05 at 555 nm	7.9	49.7

The GaN/PSi heterojunction photodiodes fabricated using the PLD process at different substrate temperatures exhibit superior performance characteristics in the UV-A band (370 nm) and yellow band (575 nm). The spectral responsivity of the photodiodes is measured to be 29.03 A/W and 19.86 A/W, with detectivity values of 8.6 × 10^12^ and 8.9 × 10^12^ Jones, and external quantum efficiency values of 97.2% and 50.89%, respectively. These photodiodes also demonstrate fast response times, with a rise time of 363 μsec and a fall time of 711 μs. These performance metrics surpass those achieved by Jiang et al. (2022), who fabricated a GaN/Si UV photodetector using a chemical vapor deposition process, which had a responsivity of 71.4 mA/W, detectivity of 7.1 × 10^8^ Jones, external quantum efficiency of 24.3%, and response time of 0.2/7.6 s^103,104^.

## Conclusion

The GaN/PSi nanostructures were successfully fabricated as heterojunction photodiodes using the pulsed laser deposition method with a 300 nm laser wavelength, 900 mJ laser energy, and different substrate temperatures ranging from 200 to 400 °C. All of the GaN/PSi heterojunction photodiodes exhibited prominent crystalline peaks with large crystallite sizes at 300 °C. Photoluminescence measurements revealed peaks at 368 nm and 728 nm, corresponding to energy gaps of 3.36 eV and 1.7 eV, respectively, which are in agreement with the theoretical values for GaN and PSi energy gaps. The morphological structure of the nanostructures exhibited a cauliflower-like shape, while the topography properties displayed a roughness of 20.15 nm and an average diameter of 56.31 nm. The spectral responsivity analysis demonstrated that all of the heterojunction photodiodes functioned effectively in UV-A and Green band photodetection. The responsivity generally increased with increasing substrate temperature up to 300 °C, beyond which it decreased due to the degradation in the crystalline quality of the GaN thin films. Consequently, a significantly improved UV photodetection capability was achieved through this novel laser technique. The optimal substrate temperature for maximizing the responsivity was found to be 300 °C, resulting in responsivity values of 23.36 at 370 nm and 14.46 at 550 nm. The detectivity values were measured to be 8.6 × 10^12^ and 8.9 × 10^12^ Jones, with external quantum efficiencies of 97.2% and 50.89%, respectively. The substrate temperature of 300 °C during the fabrication of GaN/PSi nanostructures had a significant impact on their electrical properties. While higher substrate temperatures (350 and 400 °C) can lead to improved crystallinity, reduced defect density, and increased carrier mobility, temperatures above 300 °C can cause degradation in the material properties due to thermal damage.

## Data Availability

Correspondence and requests for materials should be addressed to Makram A. Fakhri, Haneen D. Jabbar, and Evan T. Salim.
